# Supplementary material for: CHD7 regulates otic lineage specification and hair cell differentiation in human inner ear organoids
Source: Nat Commun. 2022 Nov 17;13:7053. doi: 10.1038/s41467-022-34759-8 (PMC9672366; doi:10.1038/s41467-022-34759-8)

Supplementary information

CHD7 regulates otic lineage specification and hair cell differentiation in human inner ear organoids

Jing Nie[1], Yoshitomo Ueda[1], Alexander J. Solivais[1], & Eri Hashino[1,2,*]

[1] Department of Otolaryngology-Head and Neck Surgery, Indiana University School of Medicine, Indianapolis, Indiana 46202, USA.

[2] Stark Neurosciences Research Institute, Indiana University School of Medicine, Indianapolis, Indiana 46202, USA.

*Corresponding Author

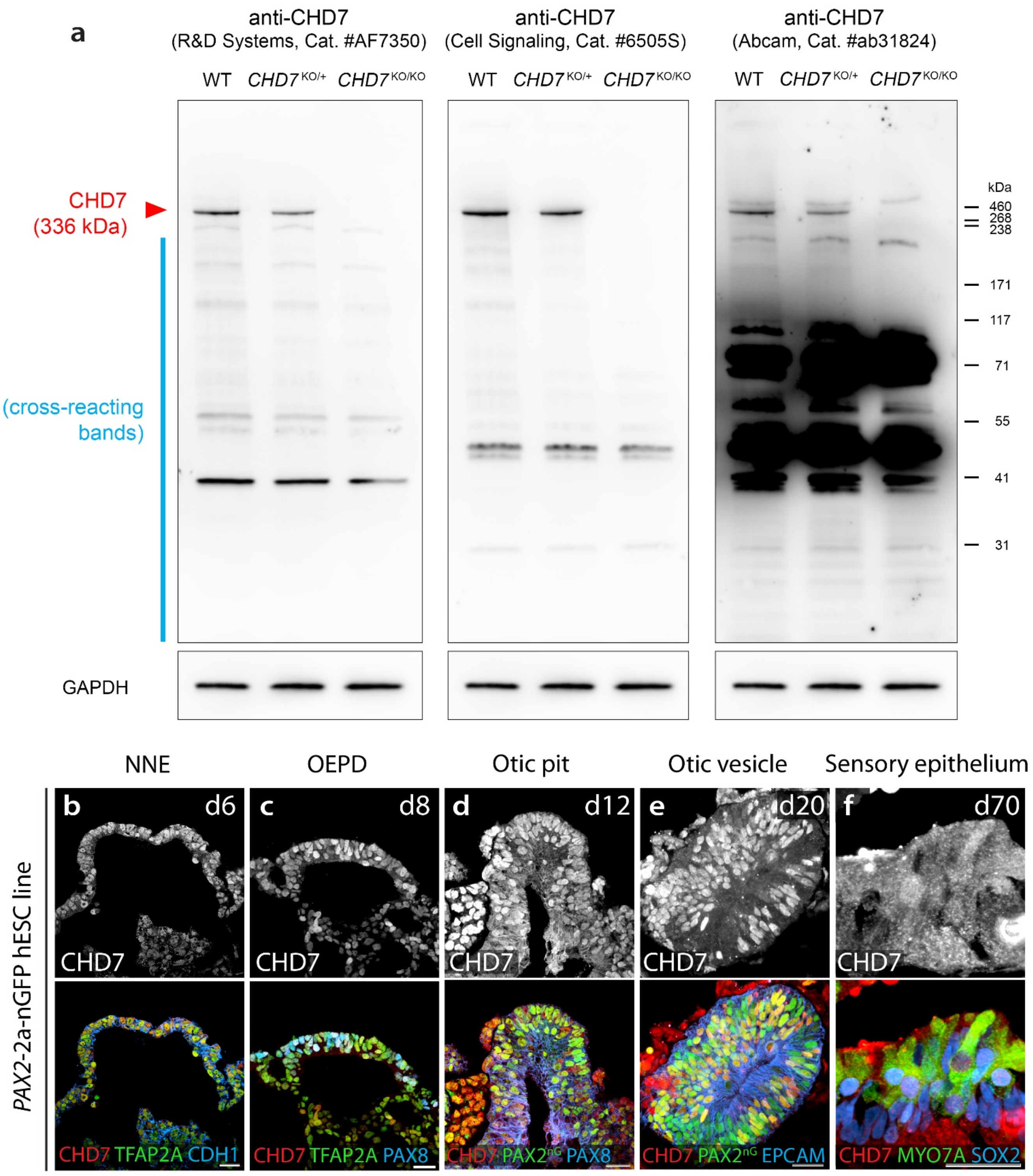
a
anti-CHD7
(R&D Systems, Cat. #AF7350)
anti-CHD7
(Cell Signaling, Cat. #6505S)
anti-CHD7
(Abcam, Cat. #ab31824)
WT
CHD7 KO/+
CHD7 KO/KO
CHD7
(336 kDa)
(cross-reacting bands)
kDa
460
268
238
171
117
71
55
41
31
GAPDH
NNE
OEPD
Otic pit
Otic vesicle
Sensory epithelium
PAX2-2a-nGFP hESC line
b d6
c d8
d d12
e d20
f d70
CHD7
CHD7 TFAP2A CDH1
CHD7 TFAP2A PAX8
CHD7 PAX2nG PAX8
CHD7 PAX2nG EPCAM
CHD7 MYO7A SOX2

Supplementary Figure 1. Low specificity of widely used CHD7 antibodies. **a**, Western blot of WT, *CHD7*$^{KO/+}$, and *CHD7*$^{KO/KO}$ hESCs showing high level of cross reactivities in three commercially available anti-CHD7 antibodies (R&D Systems #AF7350, Cell Signaling #6505S, and Abcam #ab31824). **b–f**, Immunostaining at key otic development stages in *PAX2*^{nG} human inner ear organoids using an anti-CHD7 antibody (R&D Systems #AF7350), as well as antibodies against NNE markers TFAP2A and CDH1, OEPD markers TFAP2A and PAX8, otic placode/pit and otic vesicle markers PAX2^{nG}, PAX8, and EPCAM, and hair cell markers MYO7A and SOX2 and supporting cell marker SOX2. Scale bars, 25 μm.

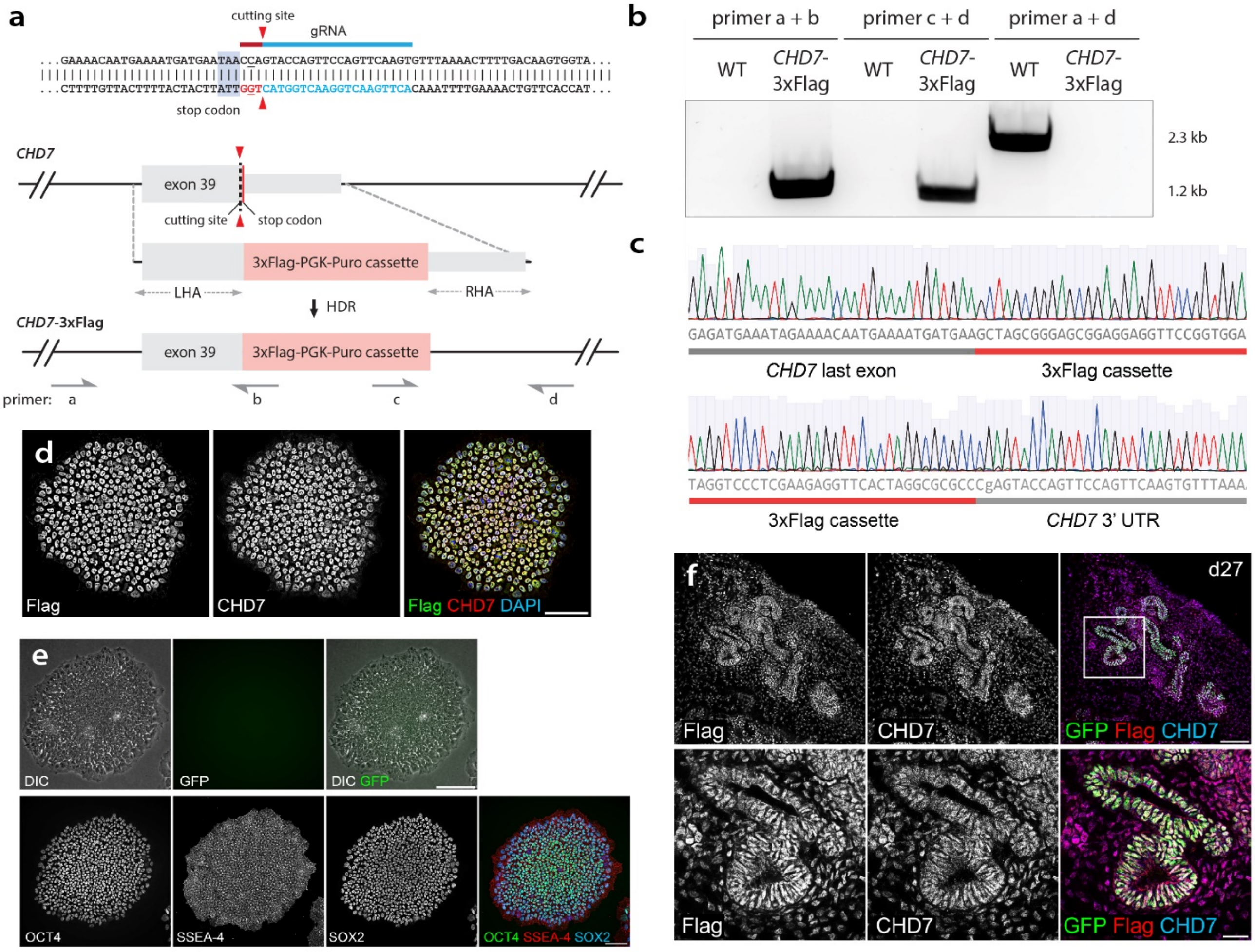

g

Off-target site	Chr.	Strand	Position	Sequence	# of mismatches	Score	Gene	Off-target indels/mutations?
1	chr19	1	9062473	ATTTGAAGTGGAACTGGTTCCAG	3	1.090163934	None	No
2	chr9	1	120867316	AATTGAAATGGAACTGGTGCTGG	3	1.090163934	None	No
3	chr5	-1	15854635	AGTTGAACTTGAACTGGTTCTGG	3	1.004040984	None	No
4	chr6	1	50108146	ATTGGAGCTTGAACTGGTACTGG	4	0.885757189	None	No
5	chr2	-1	212197528	ATTAGAAGCGGAACTGGTACTGG	4	0.846960117	None	No
6	chr1	-1	154696565	ACATTAAAAGGAACTGGTACGGG	4	0.822304167	None	No
7	chr9	1	33634099	ACATGTACTGGTACTGGTACTGG	3	0.804669905	None	No
8	chr8	1	22428447	AGGTGAACGAGAACTGGTACGAG	4	0.781289715	NM_001018003	No
9	chr12	-1	103539072	AACTGAACTGGAACTGGAACTAG	3	0.647595062	None	No
10	chr14	1	65914891	GCTGGAAGTGGAGCTGGTACTAG	4	0.581724684	None	No

Supplementary Figure 2. Labeling of endogenous CHD7 with a 3×Flag tag. **a**, CRISPR knockin design of *CHD7*-3×Flag. **b**, PCR genotyping of WT and *CHD7*-3×Flag cell lines using primers shown in (**a**) suggested successful bi-allelic 3×Flag knockin. The large PGK-Puro antibiotic selection cassette was difficult to amplify, so this PCR genotyping experiment did not use a long extension time setting to attempt to amplify the entire 3×Flag-PGK-Puro insertion. **c**, Sanger sequencing chromatograms of the left and right junction areas between the genomic DNA and the 3×Flag-PGK-Puro cassette insertion. **d**, Co-localization of anti-Flag and anti-CHD7 signals in *CHD7*-3×Flag hESCs. **e**, Normal hESC morphology and absence of *PAX2*-2a-nGFP expression, as well as normal pluripotency marker (OCT4, SSEA4, and SOX2) expression in *CHD7*-3×Flag hESCs. **f**, Co-localization of anti-Flag and anti-CHD7 signals in d27 *CHD7*-3×Flag organoids. **g**, No off-target mutations were detected at the top 10 predicted off-target sites of *CHD7*-3×Flag hESCs. Scale bars, 100 μm (**d**, **e**), 25 μm (**f**).

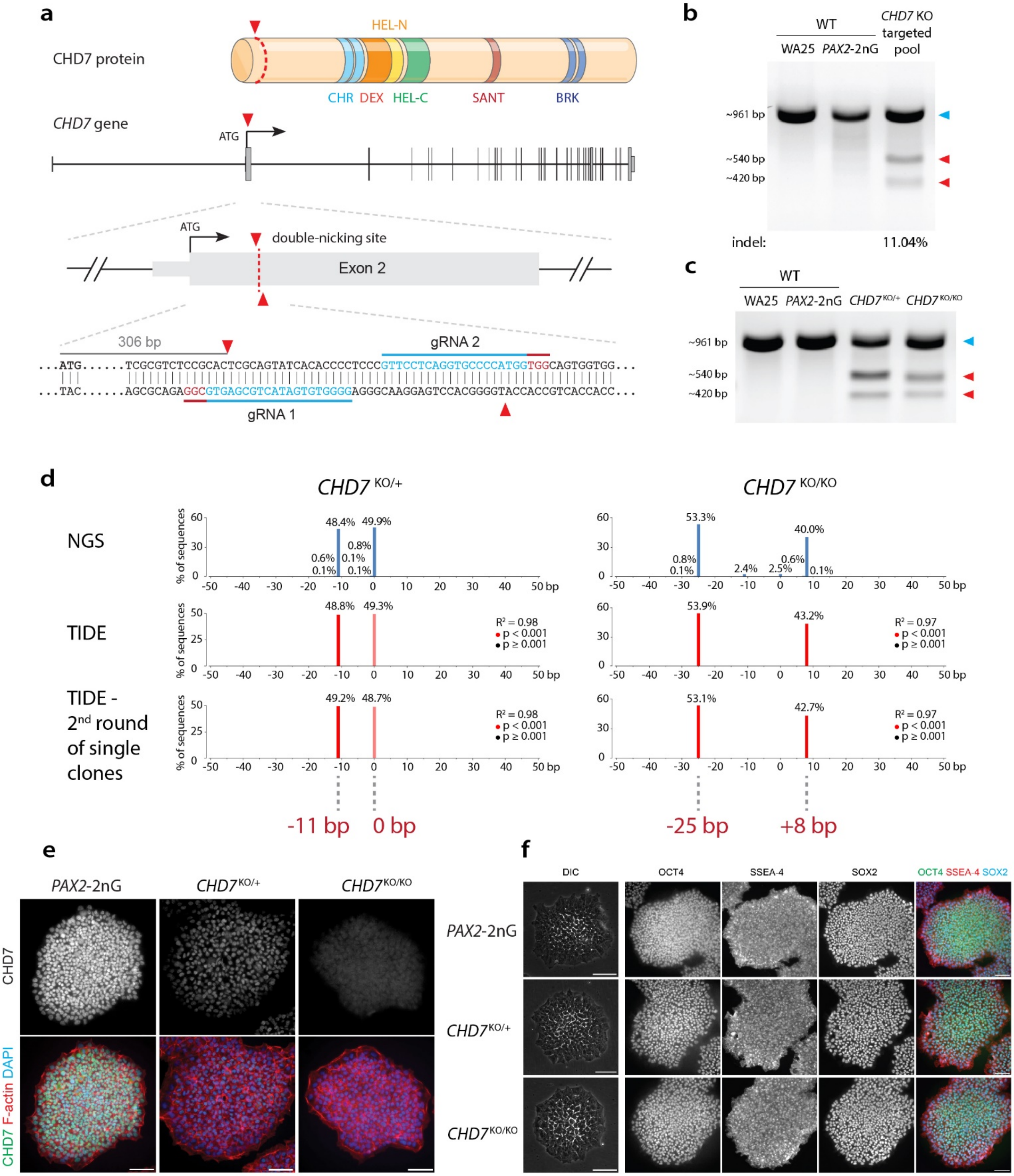

Supplementary Figure 3. Generation of *CHD7*$^{KO/+}$ and *CHD7*$^{KO/KO}$ hESC lines with CRISPR gene editing. **a**, CRISPR double nicking targeting strategy at the first coding exon of *CHD7*. **b**, T7

endonuclease 1 (T7E1) assay detected ~11.04% indel formation from *CHD7* KO targeted population of hESCs. **c**, T7E1 assay detected indel formation from *CHD7*$^{KO/+}$ and *CHD7*$^{KO/KO}$ clonal hESC lines. **d**, Next generation sequencing (NGS) and Tracking of Indels by DEcomposition (TIDE) analysis revealed a WT allele and a 11 bp frameshift deletion allele in the *CHD7*$^{KO/+}$ clonal hESC line, as well as a 25 bp frameshift deletion allele and an 8 bp frameshift insertion allele in the *CHD7*$^{KO/KO}$ clonal hESC line. Second round of clonal cell line isolation followed by TIDE analysis confirmed that the *CHD7*$^{KO/+}$ and *CHD7*$^{KO/KO}$ hESC lines are homogeneous cell lines. **e**, Anti-CHD7 immunostaining in *CHD7*$^{KO/+}$ and *CHD7*$^{KO/KO}$ hESC. Note that the CHD7 antibody (R&D Systems, #AF7350) is not highly specific based on western blot analysis (**Supplementary Fig. 1a**), thus resulted in moderate levels of background noise. **f**, *CHD7*$^{KO/+}$ and *CHD7*$^{KO/KO}$ lines showed normal hESC morphology and normal pluripotency marker (OCT4, SSEA4, and SOX2) expression. Scale bars, 50 μm.

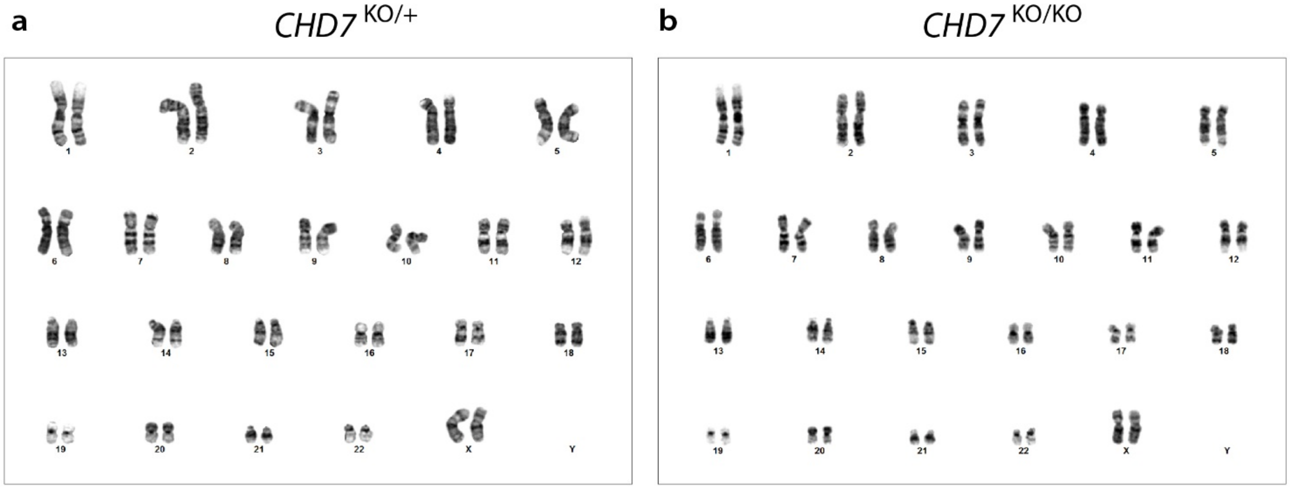

c

Off-target site	chr.	strand	position	sequence	# mismatches	score	gene	off-target indels/mutations?	
								CHD7 KO/+	*CHD7 KO/KO*
gRNA1-1	chr20	1	20337865	AGGGGGTCATACTGCGAGTGGAG	3	2.606310014	None	?	?
gRNA1-2	chr20	1	20338204	AGGGGGTCATACTGCGAGTGGAG	3	2.606310014	None	?	?
gRNA1-3	chr20	1	20336883	GAGGGGTCATACTGCGAGTGGAG	3	2.543507363	None	?	?
gRNA1-4	chr11	1	129245133	GGGCTGCGAGACTGCGAGTGGAG	3	1.599975502	None	No	No
gRNA1-5	chr5	-1	146780153	GGCTGGTGACACTGCGAGTGCAG	4	1.258804523	None	No	No
gRNA1-6	chr11	1	112829584	TGGGAGTGATACTGGGAGTGTGG	3	0.844444444	None	No	No
gRNA1-7	chr18	-1	54960416	TGTGTGTAATACTGCGTGTGTAG	4	0.611927885	None	No	No
gRNA1-8	chr7	-1	42610888	TGGGAGAGGTACTGCGAGTGTGG	4	0.58761959	None	No	No
gRNA1-9	chr6	-1	157639441	GGTGTGTGATGCTGCGAGGGGAG	3	0.577631667	None	No	No
gRNA1-10	chr9	1	131144867	AGGGAGTGAGACTGCGAGAGAGG	4	0.485227553	None	No	No
gRNA2-1	chr15	-1	77305172	CTTCCTGAGGTGCCCCATGGAGG	2	4.569366197	None	No	No
gRNA2-2	chr19	-1	14694078	CTGCCTCAGCTGCCCCATGGTGG	3	2.48975671	NM_207390	No	No
gRNA2-3	chr19	1	3969396	GTTGCTCTGGTGCCCCATGCCAG	3	1.397354497	None	No	No
gRNA2-4	chr8	-1	140655071	GCTGCTCAGGTTCCCCATGGGGG	3	1.384888889	None	No	No
gRNA2-5	chr2	-1	121570106	GGGCCTCAGGTCCCCCATGGTGG	3	1.365500444	None	No	No
gRNA2-6	chr1	1	206686559	ATTCCTCAGTTTCCCCATGGAGG	3	1.310427397	None	No	No
gRNA2-7	chr17	1	32058055	GATGCTCGGATGCCCCATGGAAG	4	1.296862648	None	No	No
gRNA2-8	chr15	1	41199793	GTCCATCTGTTGCCCCATGGTGG	4	1.258804523	None	No	No
gRNA2-9	chr10	1	72467883	GTTCCTCAGATGCCCTATGGAGG	2	1.059798592	None	No	No
gRNA2-10	chr22	-1	30178740	GCTCCCCAGGTGCCCCATGACAG	3	0.902714689	None	No	No

Supplementary Figure 4. Validation of the established *CHD7*$^{KO/+}$ and *CHD7*$^{KO/KO}$ hESC lines. **a**, *CHD7*$^{KO/+}$ and *CHD7*$^{KO/KO}$ lines showed normal karyotyping results. **b**, Off-target sequencing of the top 10 predicted off-target sites of both double-nicking gRNAs in *CHD7*$^{KO/+}$ and *CHD7*$^{KO/KO}$ hESC lines. The top 3 predicted off-target sites of gRNA #1 were in a stretch of highly repetitive genomic DNA sequence region, resulting in failure of PCR amplifications despite numerous attempts using various PCR and molecular cloning strategies. These three off-target sites were far away from any coding gene, and karyotyping analysis (**a**) detected no chromosomal translocation or other chromosomal

abnormalities. Moreover, the Cas9n nickase is unlikely to induce indel formation from a single nicking on one DNA strand. Therefore, these three predicted sites are not expected to contain mutations. Even if mutation(s) exist, they are unlikely to affect *CHD7*$^{KO/+}$ and *CHD7*$^{KO/KO}$ phenotypes.

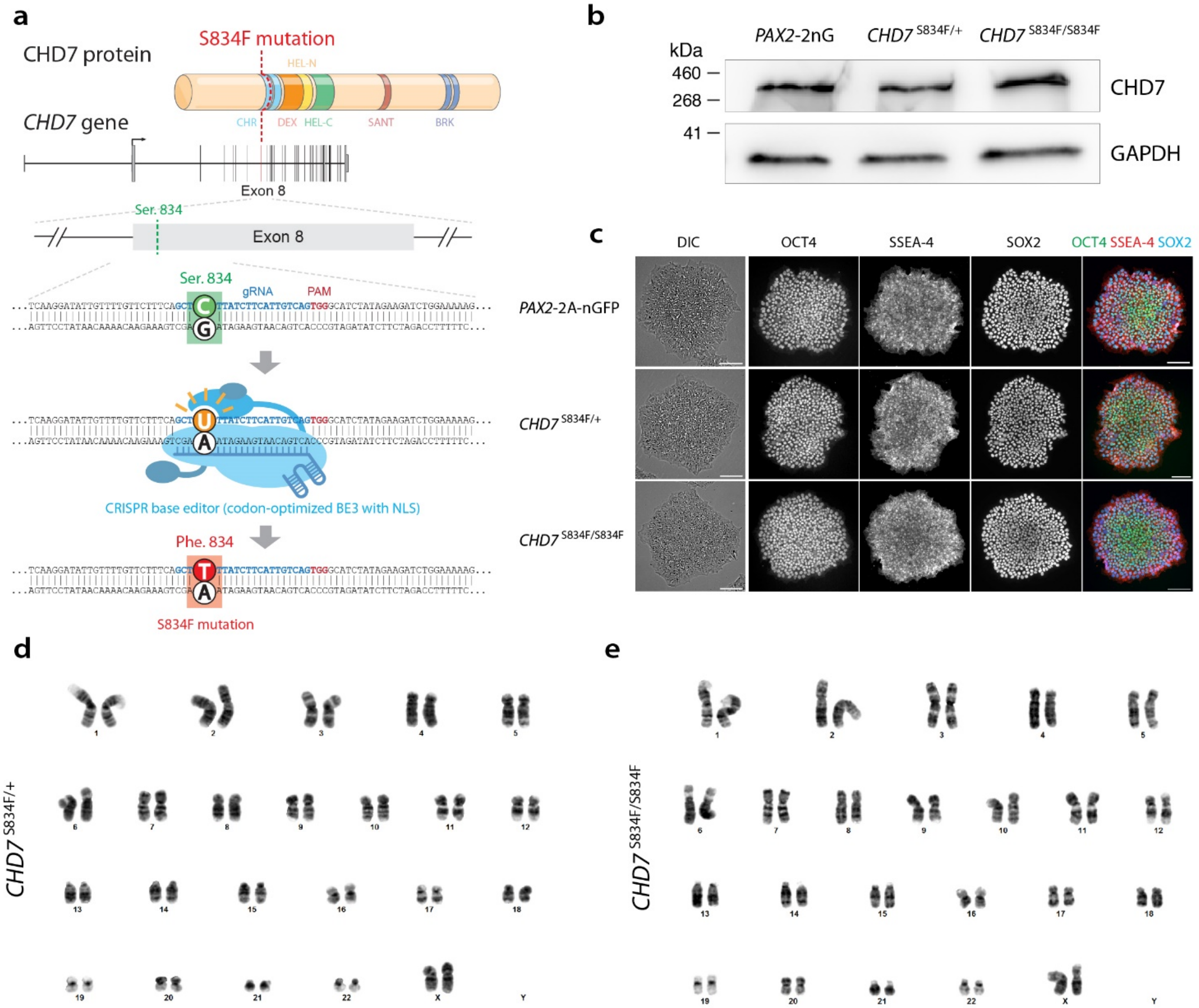

f

Off-target site	Chr.	Strand	Position	Sequence	# of mismatches	Score	Gene	Off-target mutations in the *CHD7*$^{S834F/+}$ cell line?	Off-target mutations in the *CHD7*$^{S834F/S834F}$ cell line?
1	chr3	-1	41046557	GTTCTTATCTTCATTGTCAT	2	8.611956522	None	No	No
2	chrX	-1	94295223	ACTTTTATCTTCATTGTCAG	2	5.722891566	None	No	No
3	chr7	1	38218640	GCACTTATTTTCATTGTCAG	2	4.030448592	None	No	No
4	chrX	1	17453098	AATCTTTTCTTCATTGTCAG	3	1.825175809	None	No	No
5	chrX	1	12425343	TCCCTTCTCTTCATTGTCAG	3	1.799623347	None	No	No
6	chr7	1	84144288	GCTCATTTCCTCATTGTCAG	3	1.626097551	None	No	No
7	chr10	-1	117770683	GCTAATATTTTCATTGTCAG	3	1.579455782	None	No	No
8	chr2	-1	159748105	CCGCCTACCTTCATTGTCAG	4	1.469717573	MARCH7 (ENSG00000136536)	No	No
9	chr12	-1	51981191	GCCCTTACCTTTATTGTCAG	3	1.442430047	ACVR1B (ENSG00000135503)	No	No
10	chr5	1	96962862	GCTTTTATCAACATTGTCAG	3	1.413674672	None	No	No

Supplementary Figure 5. Generation of *CHD7*$^{S834F/+}$ and *CHD7* $^{S834F/S834F}$ hESC lines with a CRISPR base editor. **a**, CRISPR base editing strategy for inducing the C to T mutation at the *CHD7* Ser. 834 locus. **b**, Western blot of WT (*PAX2*^{nG}), *CHD7*$^{S834F/+}$, and *CHD7*$^{S834F/S834F}$ hESCs suggested that protein expression levels were not affected. **c**, *CHD7*$^{S834F/+}$ and *CHD7*$^{S834F/S834F}$ lines showed normal hESC morphology and normal pluripotency marker (OCT4, SSEA4, and SOX2) expression. **d–e**, *CHD7*$^{S834F/+}$ and *CHD7*$^{S834F/S834F}$ lines showed normal karyotyping results. **f**, No mutations were found from the top 10 predicted off-target sites in the *CHD7*$^{S834F/+}$ and *CHD7*$^{S834F/S834F}$ hESC lines. Scale bars, 50 μm.

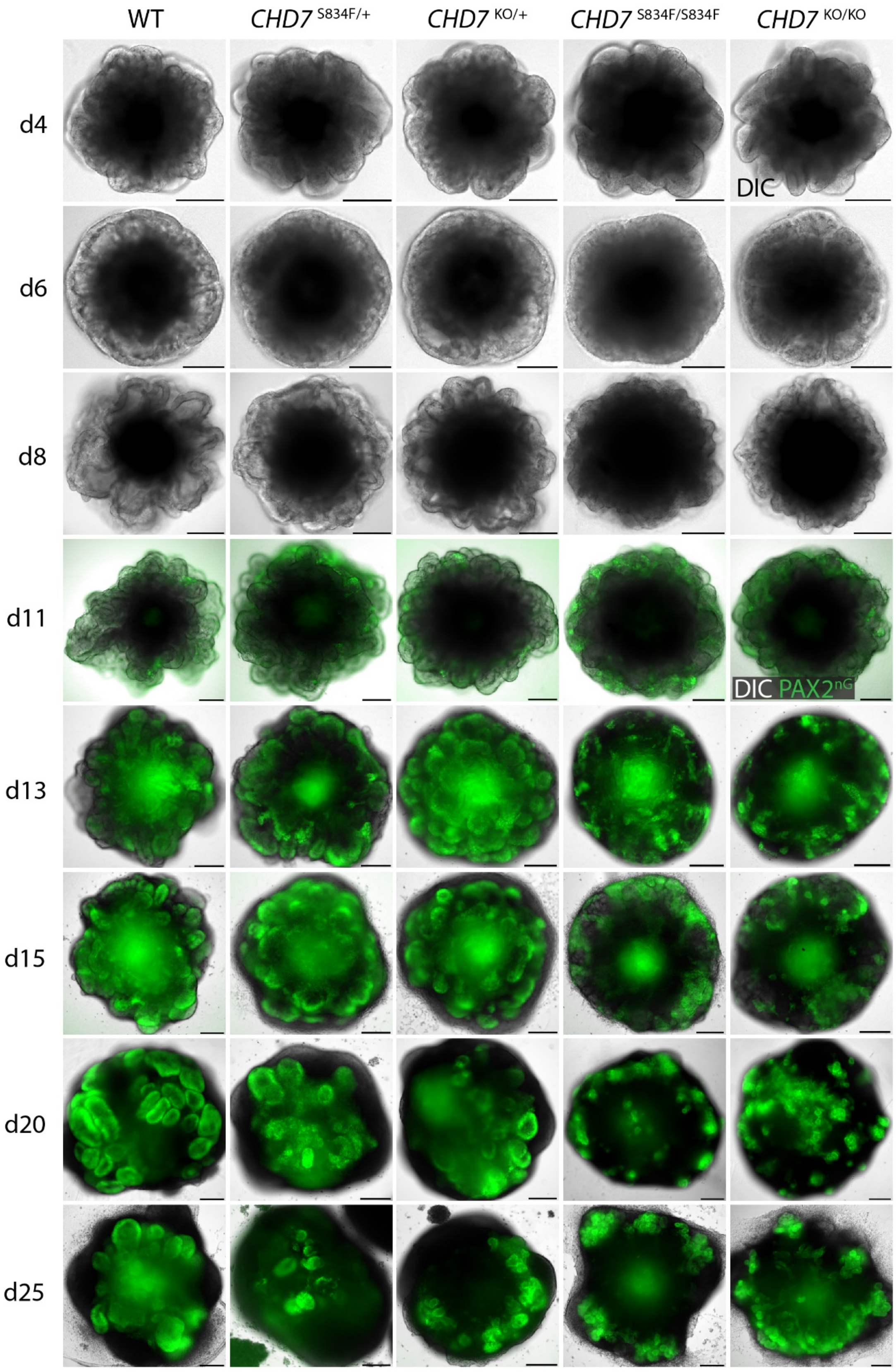
WT
CHD7 S834F/+
CHD7 KO/+
CHD7 S834F/S834F
CHD7 KO/KO
d4
d6
d8
d11
d13
d15
d20
d25
DIC
DIC PAX2nG

Supplementary Figure 6. Morphology and *PAX2*^{nG} fluorescence signals of WT (*PAX2*^{nG}), *CHD7*$^{S834F/+}$, *CHD7*$^{KO/+}$, *CHD7*$^{S834F/S834F}$, and *CHD7*$^{KO/KO}$ inner ear organoids. D4–d8 organoids were DIC live imaged only, and d11–d25 organoids were live imaged in both DIC and GFP channels. Scale bars, 250 μm.

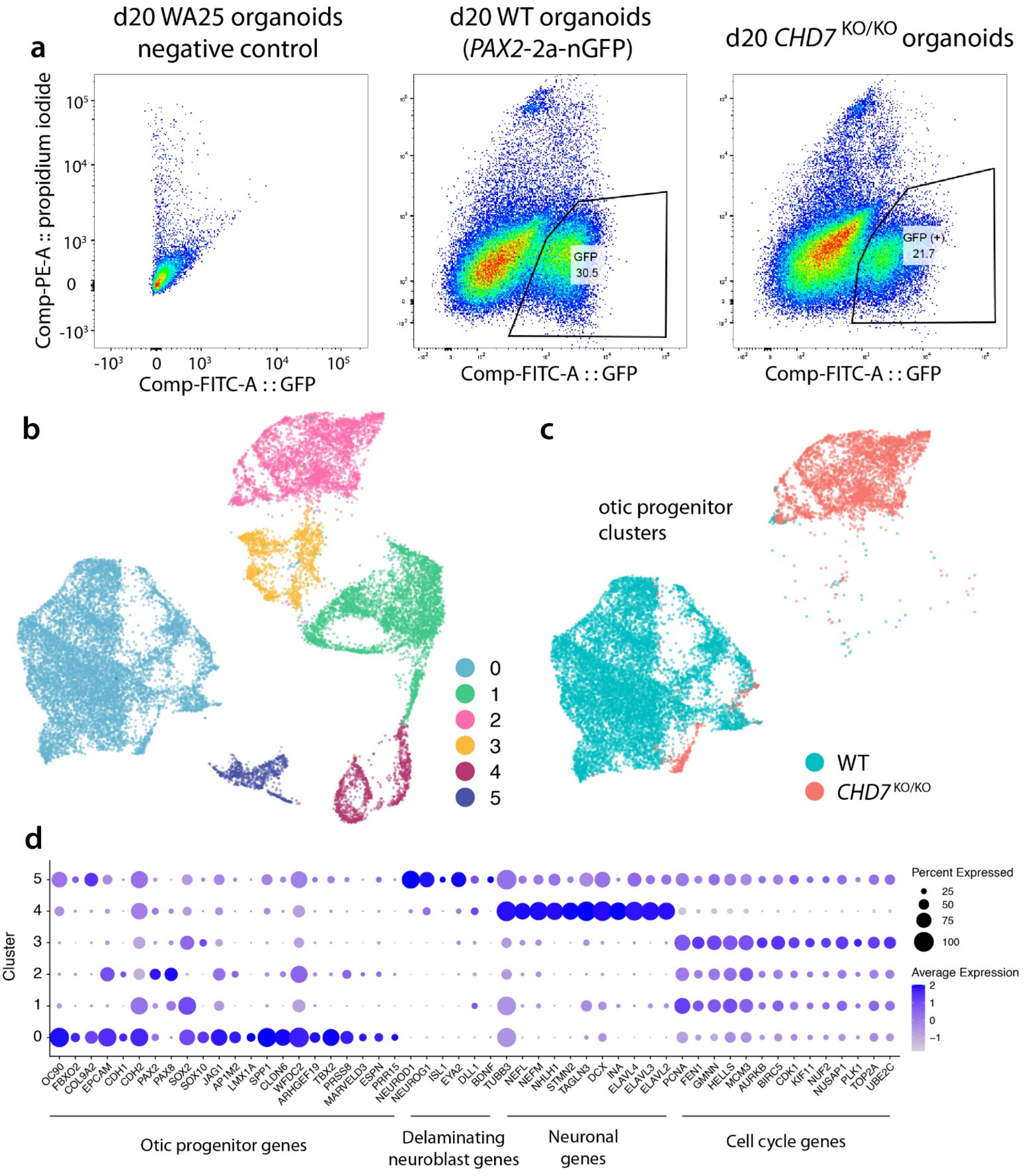

Supplementary Figure 7. scRNA-seq analysis of FACS-isolated *PAX2*^{nG} cells from d20 WT (*PAX2*^{nG}) and *CHD7*$^{KO/KO}$ inner ear organoids. **a**, FACS isolation of *PAX2*^{nG}-positive cells from d20 WT (*PAX2*^{nG}) and *CHD7*$^{KO/KO}$ inner ear organoids. **b–c**, UMAP plots of *PAX2*^{nG}-positive cells from d20 WT (*PAX2*^{nG})

and *CHD7*$^{KO/KO}$ inner ear organoids grouped by cluster (**b**) and UMAP plot of the otic progenitor subset grouped by genotype (**c**). Data represent 22,390 cells (**b**) and 14,894 cells (**c**). **d**, Dot plot of otic progenitor genes, neuroblast genes, neuronal genes, and cell cycle genes from clusters shown in (**b**). Gene expression frequency was indicated by dot size and expression level was indicated by color intensity.

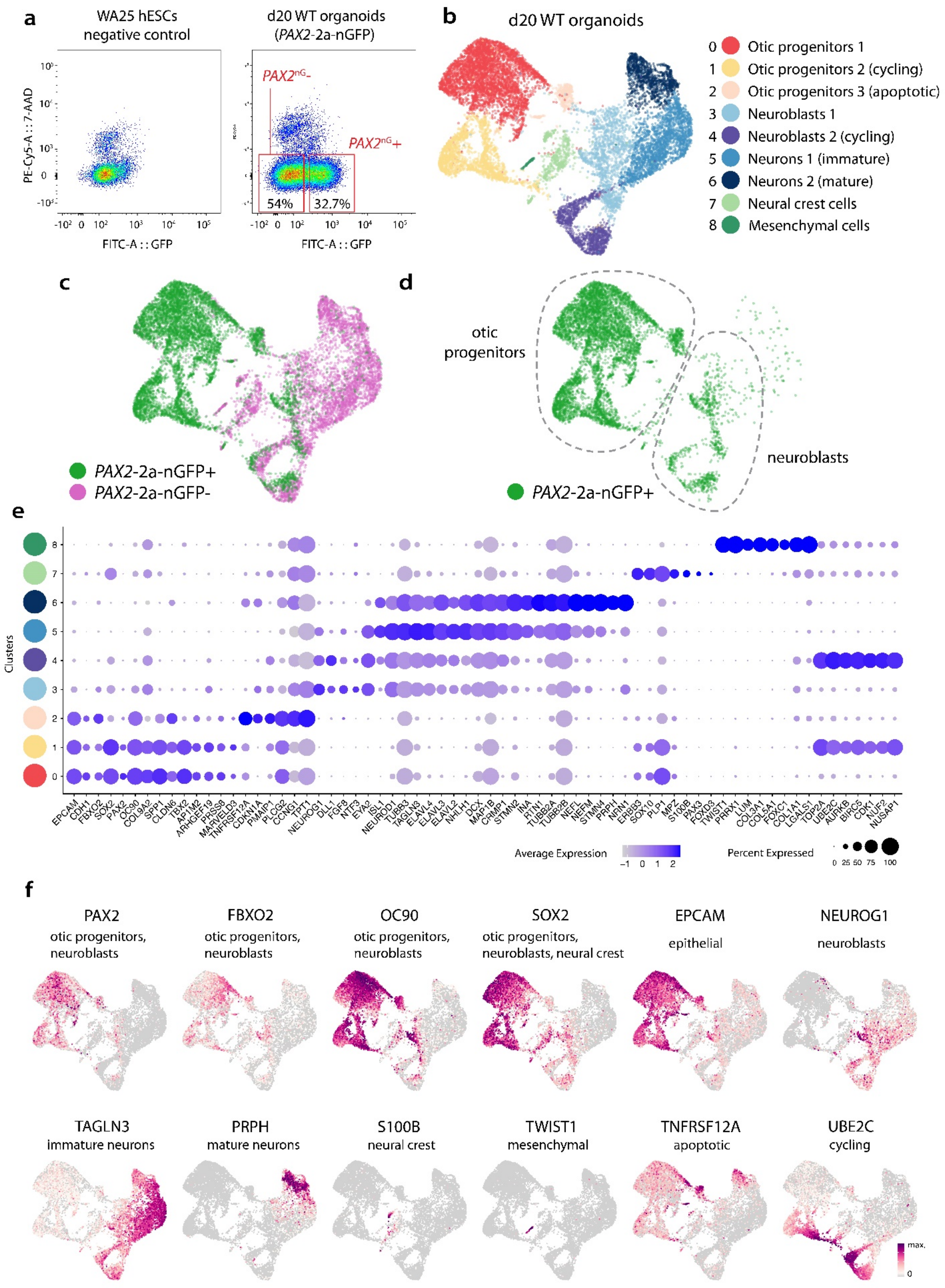
a
WA25 hESCs negative control
d20 WT organoids (PAX2-2a-nGFP)
PAX2nG-
PAX2nG+
54%
32.7%
PE-Cy5-A :: 7-AAD
FITC-A :: GFP
b
d20 WT organoids
0 Otic progenitors 1
1 Otic progenitors 2 (cycling)
2 Otic progenitors 3 (apoptotic)
3 Neuroblasts 1
4 Neuroblasts 2 (cycling)
5 Neurons 1 (immature)
6 Neurons 2 (mature)
7 Neural crest cells
8 Mesenchymal cells
c
PAX2-2a-nGFP+
PAX2-2a-nGFP-
d
otic progenitors
neuroblasts
PAX2-2a-nGFP+
e
Clusters
Average Expression
Percent Expressed
f
PAX2
otic progenitors, neuroblasts
FBXO2
otic progenitors, neuroblasts
OC90
otic progenitors, neuroblasts
SOX2
otic progenitors, neuroblasts, neural crest
EPCAM
epithelial
NEUROG1
neuroblasts
TAGLN3
immature neurons
PRPH
mature neurons
S100B
neural crest
TWIST1
mesenchymal
TNFRSF12A
apoptotic
UBE2C
cycling
max.
0

Supplementary Figure 8. scRNA-seq analysis of FACS-isolated *PAX2*^{nG}+ and *PAX2*^{nG}- cells from d20 WT (*PAX2*^{nG}) inner ear organoids. **a**, FACS isolation of *PAX2*^{nG}-positive and *PAX2*^{nG}-negative cells from d20 WT (*PAX2*^{nG}) organoids. **b**-**c**, UMAP plot of merged *PAX2*^{nG}+ and *PAX2*^{nG}- populations grouped by cluster (**b**) or by FACS groups (**c**). Data represents 13,059 total cells. (**d**) shows the UMAP plot of the FACS-isolated *PAX2*^{nG}+ population (6,995 cells), which mainly contains otic progenitors and neuroblast cells. **e**-**f**, Dot plot (**e**) and feature plots (**f**) showing marker genes of the annotated clusters. Gene expression frequency was indicated by dot size and expression level was indicated by color intensity in (**e**), and the color bar in (**f**) shows the log-normalized expression scale.

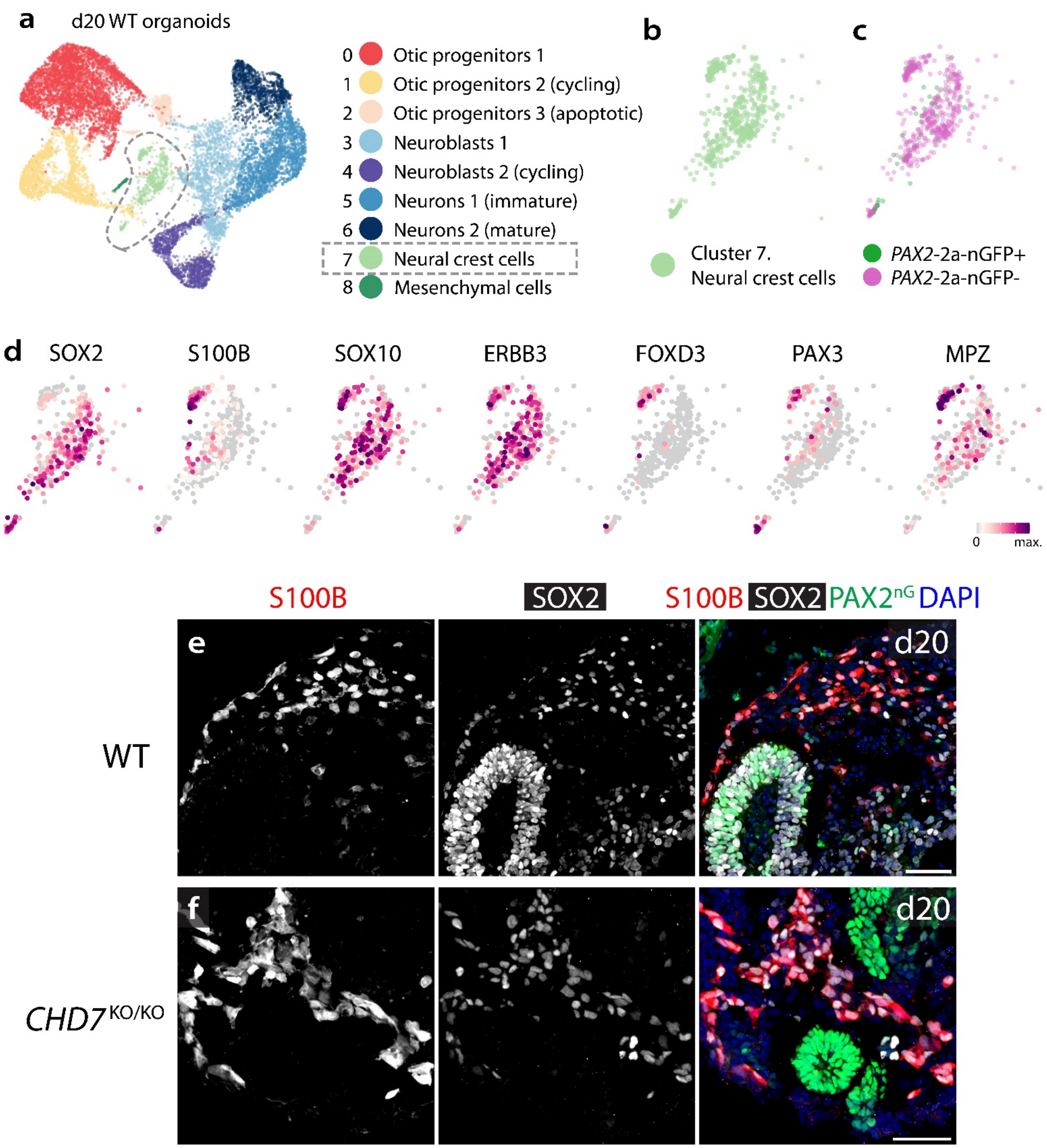

Supplementary Figure 9. scRNA-seq and immunofluorescence analysis of the neural crest cell population in d20 organoids. **a**, UMAP plot of d20 WT (*PAX2*^{nG}) inner ear organoid cells highlighting the neural crest cell cluster (dotted line). **b-c**, Bioinformatically isolated Cluster 7 cells grouped by cluster (**b**) or by FACS populations (**c**). **d**, Feature plots showing neural crest markers *SOX2*, *S100B*,

SOX10, *ERBB3*, *FOXD3*, *PAX3*, and *MPZ* expression in Cluster 7. The color bar shows the log-normalized expression scale. **e**-**f**, Immunostaining of d20 WT (**e**) and *CHD7*$^{KO/KO}$ (**f**) organoids, showing co-expression of SOX2+ S100B+ neural crest cells outside of *PAX2*^{nG}+ otic vesicles. Scale bars, 50 µm.

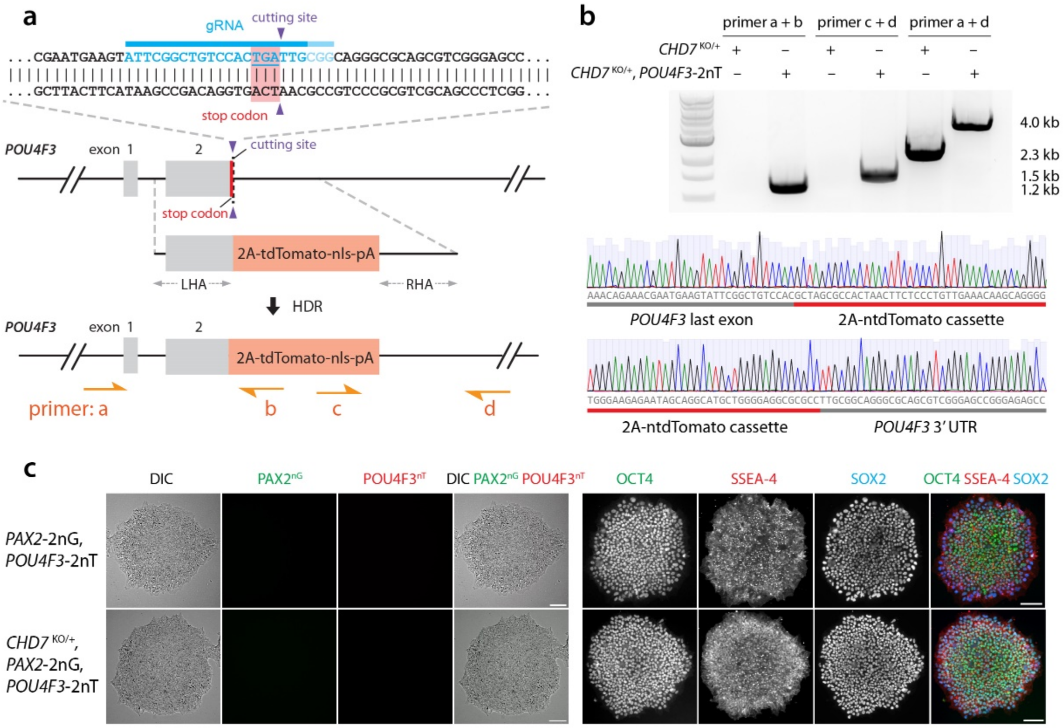

d

Off-target site	Chr.	Strand	Position	Sequence	# of mismatches	Score	Gene	Off-target mutations?
1	chr2	1	16391400	GTTCTGCTTTCCACTGATTGGAG	3	1.632771	None	No
2	chr3	-1	188314251	AGTTGTCTGTCCACTGATTGTAG	3	1.468072	None	No
3	chr12	-1	101614310	TCTCTGCTGTCCACTGATTTAAG	4	0.710795	None	No
4	chr4	1	53847222	TTTCTGCGGTCCACTGATTTAAG	4	0.710795	None	No
5	chr18	1	68394376	AGTTAGCTGTCCACTGATTCAGG	4	0.697447	None	No
6	chr11	1	128463498	ATGCAGCAGTCCACTGATTACAG	4	0.675007	None	No
7	chr2	1	152741440	AATCTGCTGTCCACTGAGTGGGG	3	0.65679	None	No
8	chr8	1	93202010	GGTCTGCTGTCCGCTGATTGTGG	4	0.581725	None	No
9	chr1	-1	194082508	TTTCTGCTGGCCACTGGTTGAAG	4	0.571588	None	No
10	chr9	-1	8278749	TTTCTTATGTCCACTGATTGGAG	4	0.564015	None	No

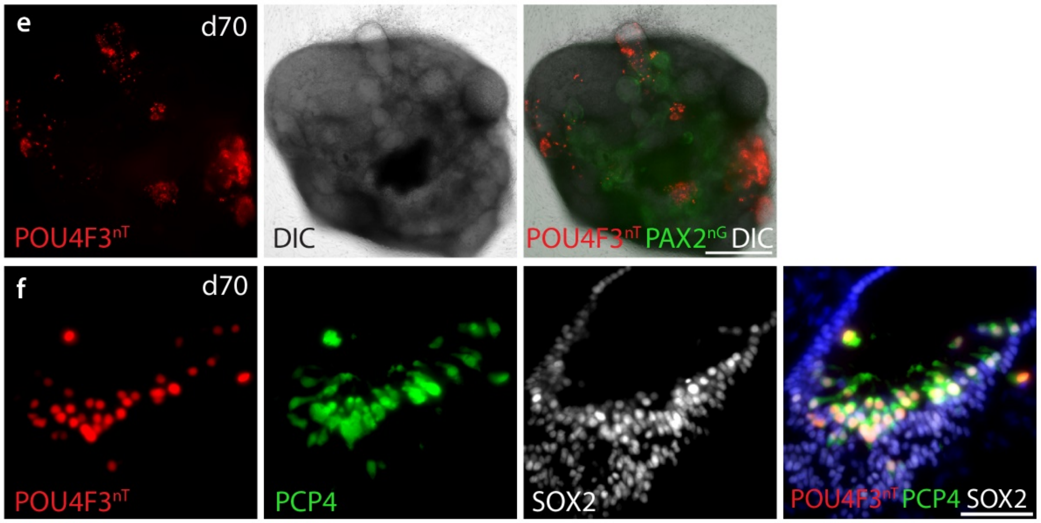

Supplementary Figure 10. Generation of *POU4F3*-2a-ntdTomato (*POU4F3*^{nT}) knockin hESC line on the *CHD7*$^{KO/+}$ genetic background. **a**, CRISPR genome engineering design of 2a-ntdTomato cassette knockin to the *POU4F3* stop codon locus. **b**, Genotyping PCR and sequencing of the *CHD7*$^{KO/+}$ *POU4F3*^{nT} hESC line using primers shown in (**a**) suggested successful bi-allelic 2a-ntdTomato knockin at the *POU4F3* stop codon locus. **c**, The *CHD7*$^{KO/+}$ *POU4F3*^{nT} line showed normal hESC morphology, normal pluripotency marker expression (OCT4, SSEA4, and SOX2), and absence of *PAX2*^{nG} or *POU4F3*^{nT} fluorescence signals in hESCs. **d**, No off-target mutations were detected from the top 10 predicted off-target sites. **e**, Live imaging of d70 *CHD7*$^{KO/+}$ *POU4F3*^{nT} organoids, showing *POU4F3*^{nT} fluorescence signals from *CHD7*$^{KO/+}$ hair cells. **f**, Immunostaining of the hair cell markers PCP4, the hair cell and supporting cell marker SOX2, as well as POU4F3^{nT} fluorescence in d70 *CHD7*$^{KO/+}$ *POU4F3*^{nT} organoids. Scale bars, 500 μm (**e**), 50 μm (**f**).

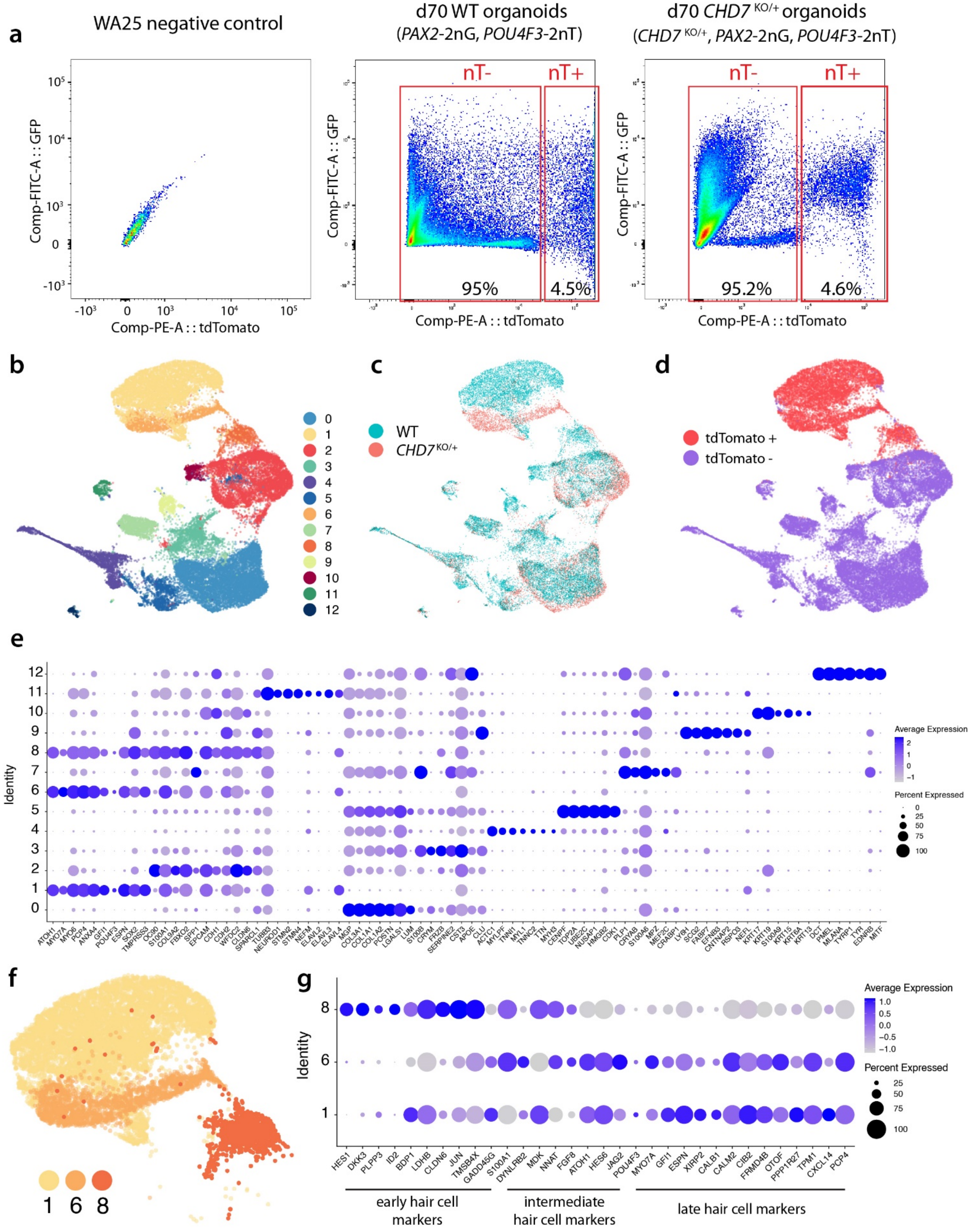
a
WA25 negative control
d70 WT organoids
(PAX2-2nG, POU4F3-2nT)
d70 CHD7 KO/+ organoids
(CHD7 KO/+, PAX2-2nG, POU4F3-2nT)
Comp-FITC-A : : GFP
Comp-PE-A : : tdTomato
nT-
nT+
95%
4.5%
95.2%
4.6%
b
c
d
WT
CHD7 KO/+
tdTomato +
tdTomato -
e
Identity
Average Expression
Percent Expressed
f
1 6 8
g
Identity
HES1 DKK3 PLPP3 ID2 BDP1 LDHB CLDN6 JUN TMSB4X GADD45G S100A1 DYNLRB2 MDK NNAT FGF8 ATOH1 HES6 JAG2 POU4F3 MYO7A GFI1 ESPN XIRP2 CALB1 CALM2 CIB2 FRMD4B OTOF PPP1R27 TPM1 CXCL14 PCP4
early hair cell markers
intermediate hair cell markers
late hair cell markers
Average Expression
Percent Expressed

Supplementary Figure 11. scRNA-seq analysis of FACS-isolated *POU4F3*^{nT}-positive and -negative cells from micro-dissected d70 WT (*POU4F3*^{nT} *PAX2*^{nG}) and *CHD7*$^{KO/+}$ (*CHD7*$^{KO/+}$ *POU4F3*^{nT} *PAX2*^{nG}) inner ear organoids. **a**, FACS isolation of *POU4F3*^{nT}-positive and -negative cells from micro-dissected d70 WT and *CHD7*$^{KO/+}$ inner ear organoids. **b–d**, UMAP plots of merged datasets of d70 WT and *CHD7*$^{KO/+}$ inner ear organoids plotted by cluster (**b**), by genotype (**c**), or by FACS isolation (**d**). **e**, Dot plot of marker genes and highly expressed genes from each cluster shown in (**e**). Gene expression frequency was indicated by dot size and expression level was indicated by color intensity. **f**, Subsetting of the three hair cell clusters. **g**, Dot plot of early, intermediate, and late hair cell marker genes from each cluster shown in (**f**).

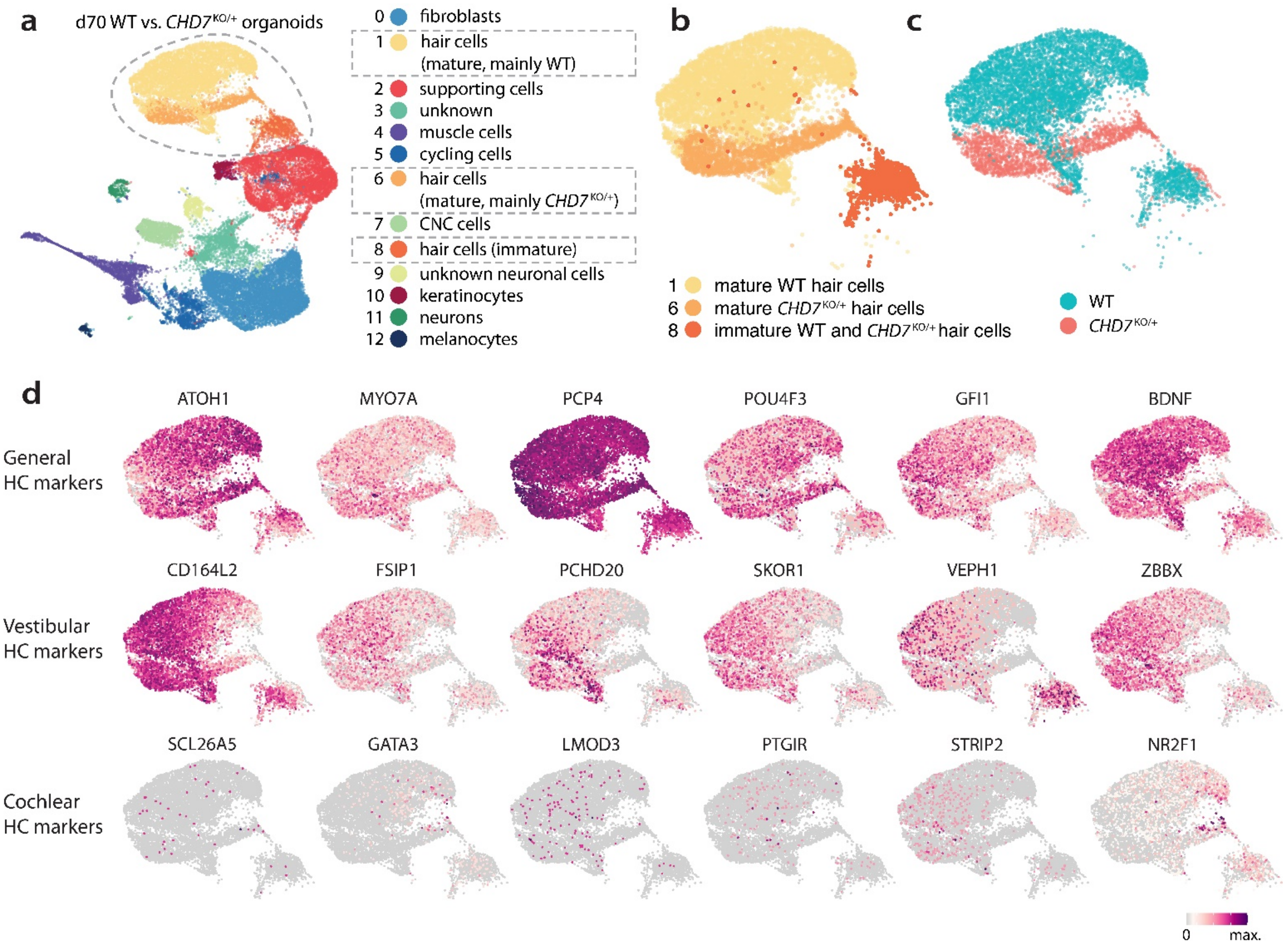

Supplementary Figure 12. scRNA-seq analysis of the hair cell population in d70 organoids. **a**–**c**, UMAP plots of merged datasets of d70 WT and *CHD7*$^{KO/+}$ inner ear organoids. The hair cell clusters highlighted in (**a**) (dotted line) was bioinformatically isolated and was plotted by cluster (**b**) or by genotype (**c**). **d**, Feature plots showing the expression of general hair cell markers (ATOH1, MYO7A, PCP4, POU4F3, GFI1, and BDNF), vestibular hair cell markers (CD164L2, FSIP1, PCHD20, SKOR1, VEPH1, and ZBBX), and cochlear hair cell markers (SLC26A5, GATA3, LMOD3, PTGIR, STRIP2, and NR2F1). The color bar shows the log-normalized expression scale.

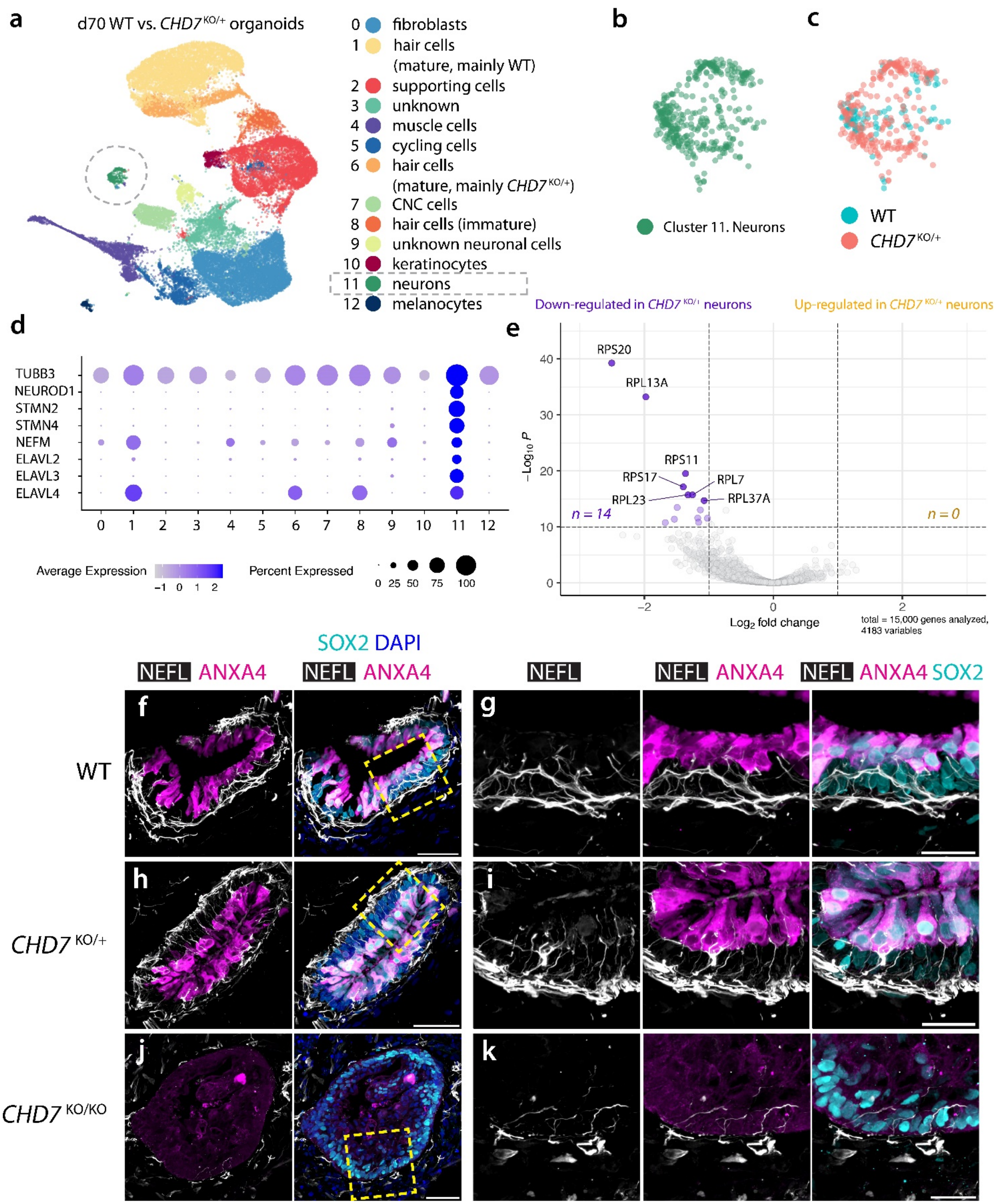

Supplementary Figure 13. scRNA-seq and immunofluorescence analysis of the neuron population in d70 organoids. **a**–**c**, UMAP plots of merged datasets of d70 WT and *CHD7*$^{KO/+}$ inner ear organoids. The

neuron cluster highlighted in (**a**) (dotted line) was bioinformatically isolated and was plotted by cluster (**b**) or by genotype (**c**). **d**, Dot plot showing neuronal marker gene expression among clusters. **e**, Volcano plot of differentially expressed genes in *CHD7*$^{KO/+}$ neurons compared to WT neurons. **f–k**, Immunofluorescence of d70 WT (**f**, **g**), *CHD7*$^{KO/+}$(**h**, **i**), and *CHD7*$^{KO/KO}$ (**j**, **k**) organoids using neuron marker NEFL, hair cell marker ANXA4, and hair cell and supporting cell marker SOX2. **g**, **i**, and **k** are enlarged views of the dotted box region shown in **f**, **h**, and **j**, respectively. For DE analysis in (**e**), the two-sided test DESeq2 was used, and adjustments were made by the Benjamini-Hochberg method for multiple comparisons. Scale bars, 50 μm.

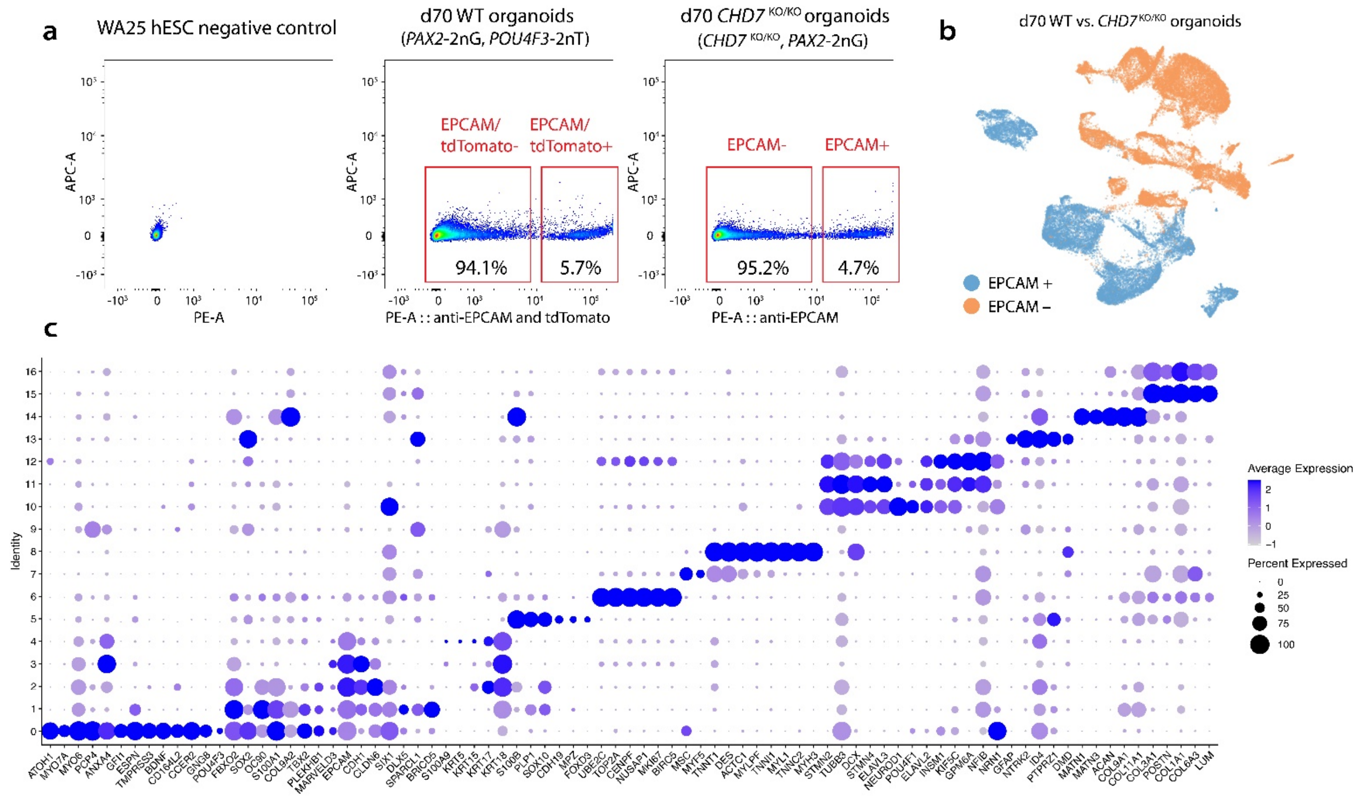

Supplementary Figure 14. scRNA-seq analysis of FACS-isolated EPCAM+ and EPCAM- cells from micro-dissected d70 WT (*POU4F3*^{nT} *PAX2*^{nG}) and *CHD7*$^{KO/KO}$ (*CHD7*$^{KO/KO}$ *PAX2*^{nG}) organoids. **a**, FACS isolation of EPCAM+ and EPCAM- cells from micro-dissected d70 WT and *CHD7*$^{KO/KO}$ inner ear organoids. The *POU4F3*^{nT}+ hair cells in WT organoids endogenously emit tdTomato fluorescence. These hair cells were also stained positive for PE-conjugated EPCAM, making them double-positive for these two red fluorescence signals. The *POU4F3*^{nT} reporter is highly specific to hair cells and are not present in the EPCAM- populations. **b**, UMAP plot of d70 WT and *CHD7*$^{KO/KO}$ organoids grouped by EPCAM+ or EPCAM- FACS identities. **c**, Dot plot of marker genes and highly expressed genes of each cluster as shown in Fig. 6a. Gene expression frequency was indicated by dot size and expression level was indicated by color intensity.

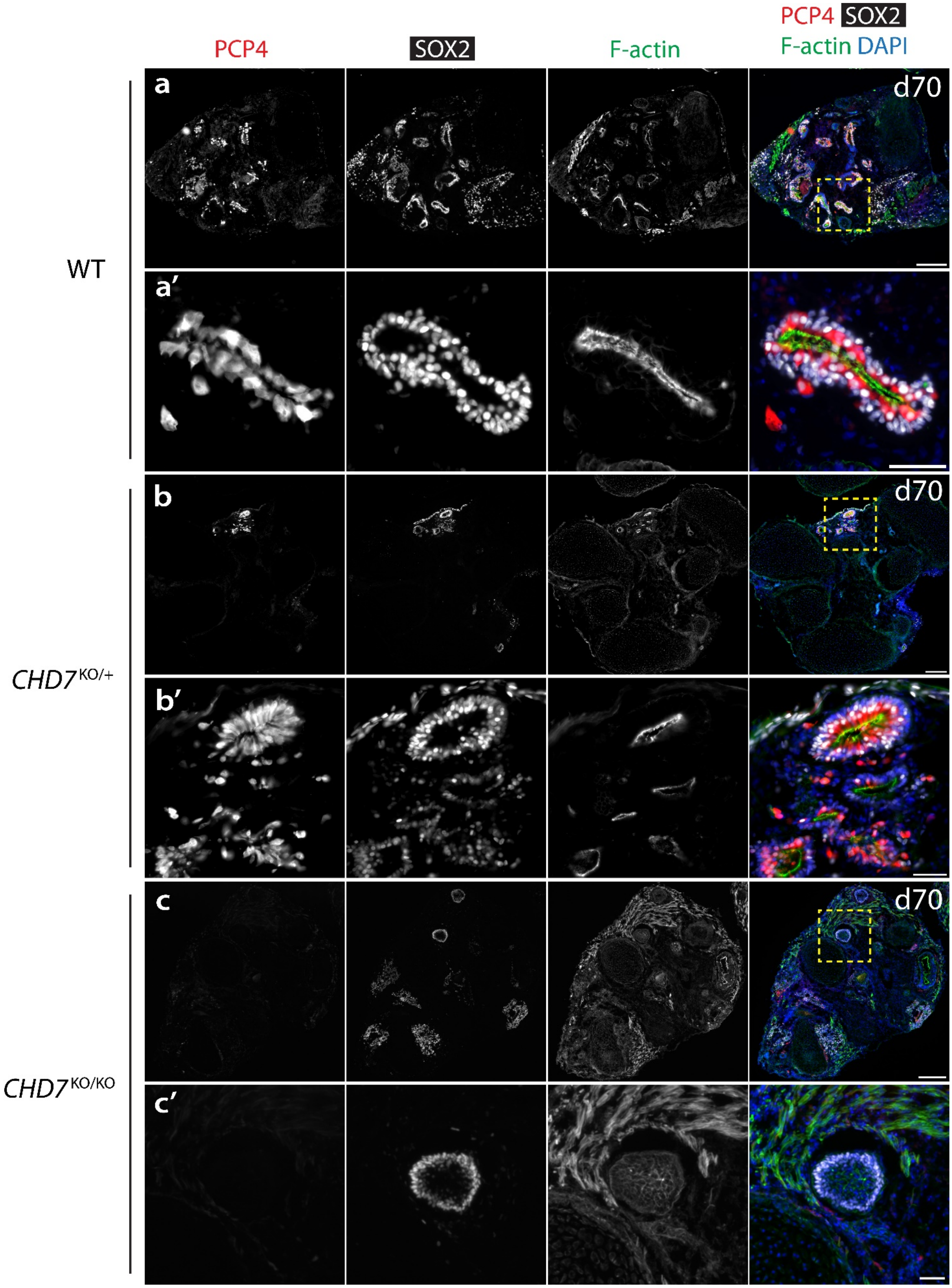
PCP4
SOX2
F-actin
PCP4 SOX2
F-actin DAPI
WT
a
d70
a'
b
d70
b'
c
d70
c'
CHD7 KO/+
CHD7 KO/KO

Supplementary Figure 15. d70 *CHD7*$^{KO/KO}$ organoids fail to generate hair cells. **a**–**c**, Low magnification (**a**, **b**, **c**) and high magnification (**a'**, **b'**, **c'**) immunofluorescence images of d70 WT (**a**, **a'**), *CHD7*$^{KO/+}$ (**b**, **b'**), and *CHD7*$^{KO/KO}$ (**c**, **c'**) organoids. Antibodies highlight hair cells (PCP4), SOX2 (otic epithelial cells, including supporting cells and hair cells), and F-actin (high intensity in stereocilia of hair cells). Scale bars, 250 µm (**a**, **b**, **c**), and 50 µm (**a'**, **b'**, **c'**).

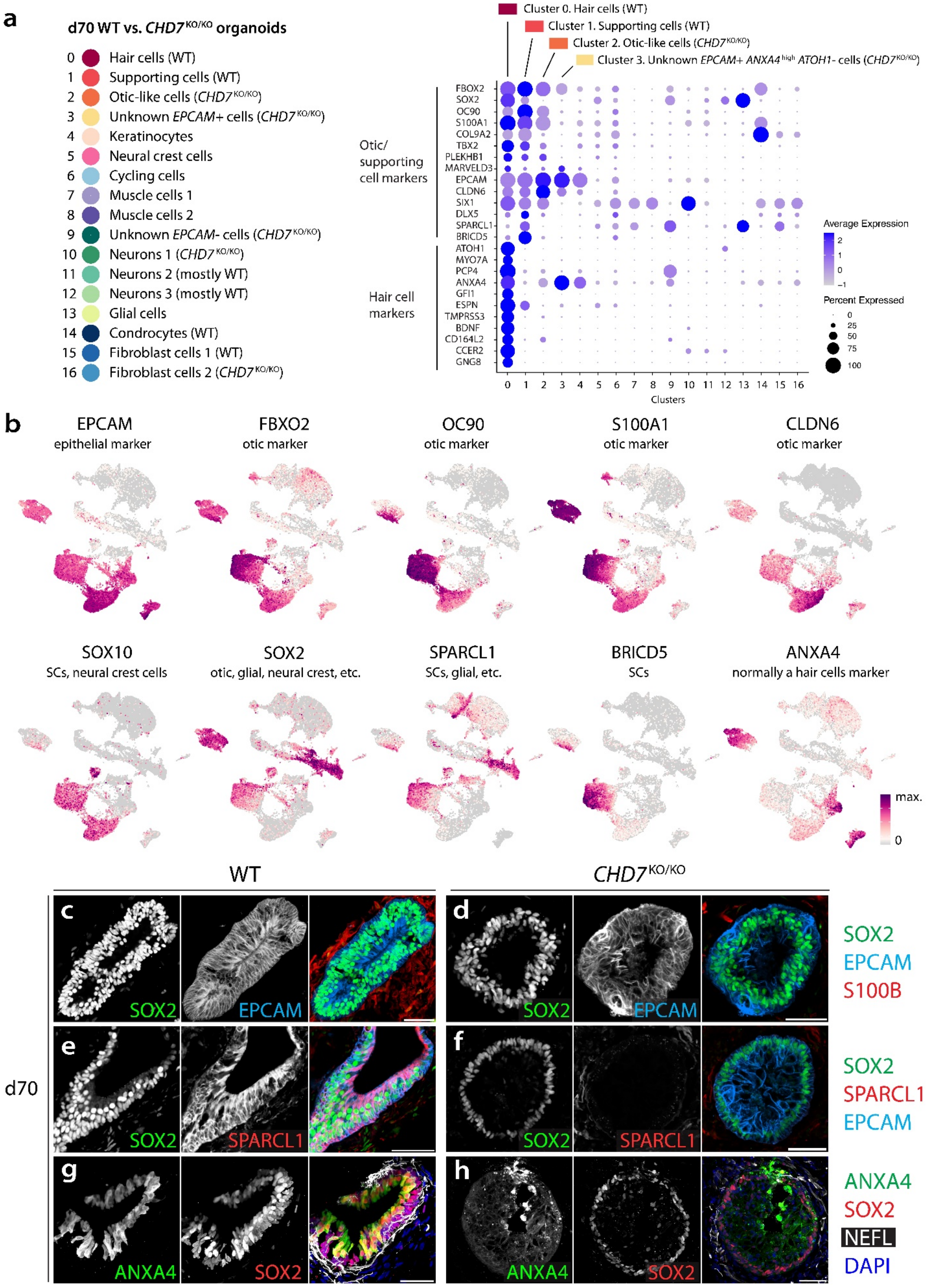

a
d70 WT vs. CHD7 KO/KO organoids
0 Hair cells (WT)
1 Supporting cells (WT)
2 Otic-like cells (CHD7 KO/KO)
3 Unknown EPCAM+ cells (CHD7 KO/KO)
4 Keratinocytes
5 Neural crest cells
6 Cycling cells
7 Muscle cells 1
8 Muscle cells 2
9 Unknown EPCAM- cells (CHD7 KO/KO)
10 Neurons 1 (CHD7 KO/KO)
11 Neurons 2 (mostly WT)
12 Neurons 3 (mostly WT)
13 Glial cells
14 Condrocytes (WT)
15 Fibroblast cells 1 (WT)
16 Fibroblast cells 2 (CHD7 KO/KO)
Cluster 0. Hair cells (WT)
Cluster 1. Supporting cells (WT)
Cluster 2. Otic-like cells (CHD7 KO/KO)
Cluster 3. Unknown EPCAM+ ANXA4 high ATOH1- cells (CHD7 KO/KO)
Otic/ supporting cell markers
Hair cell markers
FBOX2
SOX2
OC90
S100A1
COL9A2
TBX2
PLEKHB1
MARVELD3
EPCAM
CLDN6
SIX1
DLX5
SPARCL1
BRICD5
ATOH1
MYO7A
PCP4
ANXA4
GFI1
ESPN
TMPRSS3
BDNF
CD164L2
CCER2
GNG8
0 1 2 3 4 5 6 7 8 9 10 11 12 13 14 15 16
Clusters
Average Expression
2
1
0
-1
Percent Expressed
0
25
50
75
100
b
EPCAM
epithelial marker
FBXO2
otic marker
OC90
otic marker
S100A1
otic marker
CLDN6
otic marker
SOX10
SCs, neural crest cells
SOX2
otic, glial, neural crest, etc.
SPARCL1
SCs, glial, etc.
BRICD5
SCs
ANXA4
normally a hair cells marker
max.
0
WT
CHD7 KO/KO
d70
c
SOX2
EPCAM
d
SOX2
EPCAM
SOX2
EPCAM
S100B
e
SOX2
SPARCL1
f
SOX2
SPARCL1
SOX2
SPARCL1
EPCAM
g
ANXA4
SOX2
h
ANXA4
SOX2
ANXA4
SOX2
NEFL
DAPI

Supplementary Figure 16. scRNA-seq analysis of FACS-isolated EPCAM+ and EPCAM- cells from micro-dissected d70 WT (*POU4F3*^{nT} *PAX2*^{nG}) and *CHD7*$^{KO/KO}$ (*CHD7*$^{KO/KO}$ *PAX2*^{nG}) organoids. **a**, Dot plot of hair cell markers and supporting cell markers of each cluster as shown in Fig. 6a. Gene expression frequency was indicated by dot size and expression level was indicated by color intensity. **b**, Feature plot of key otic markers and supporting cell (SC) markers. The color bar shows the log-normalized expression scale. **c**–**h**, Immunofluorescence staining showing hair cells and supporting cells in d70 WT organoids and the EPCAM+ SOX2^{low} ANXA4^{low} SPARCL1- otic-like cells in d70 *CHD7*$^{KO/KO}$ organoids. Scale bars, 50 µm.

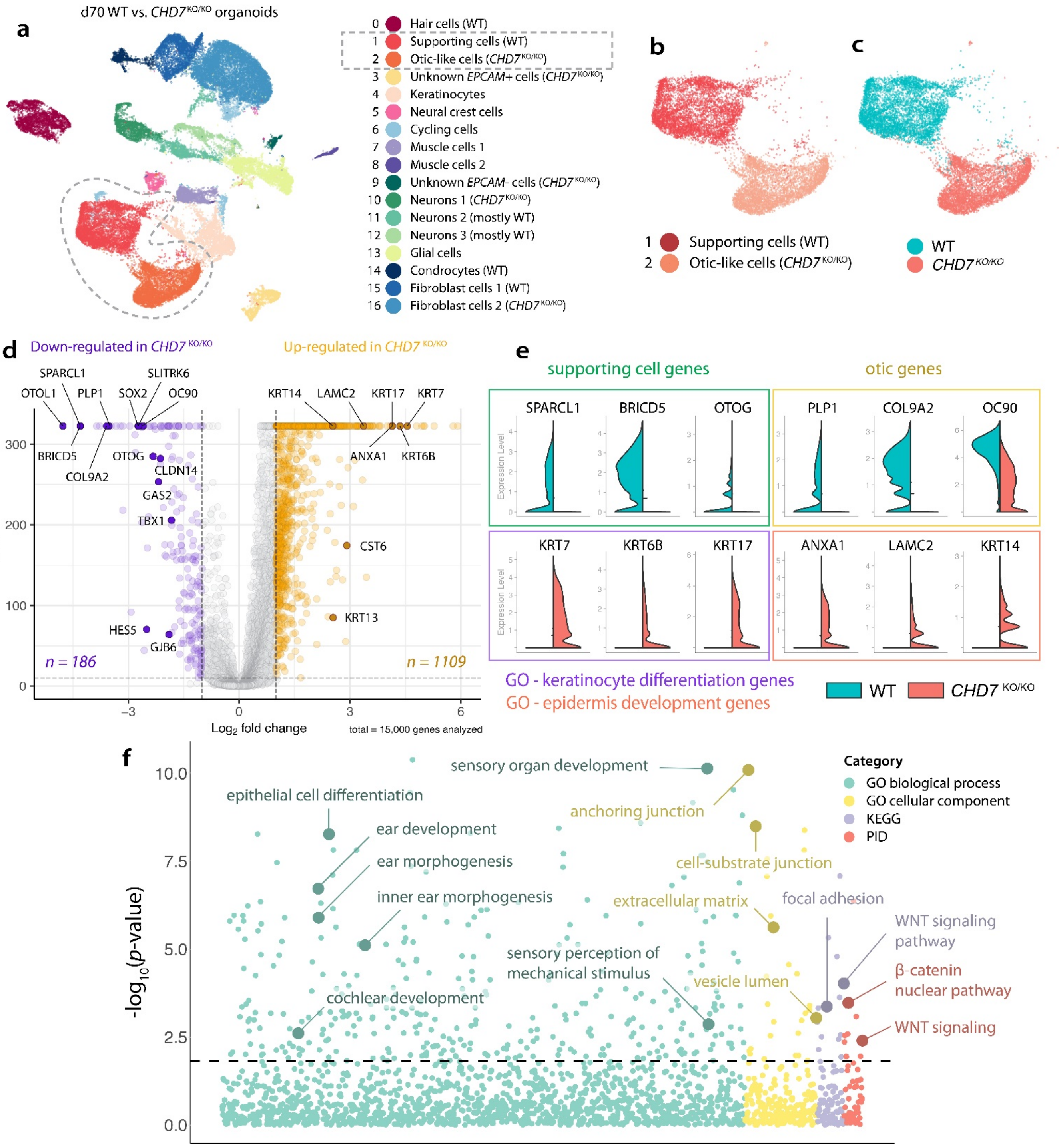

Supplementary Figure 17. scRNA-seq analysis of the supporting cell cluster from d70 WT (*POU4F3*^{nT} *PAX2*^{nG}) organoids and the otic-like cell cluster from *CHD7*$^{KO/KO}$ (*CHD7*$^{KO/KO}$ *PAX2*^{nG}) organoids. **a**, UMAP plot of d70 WT and *CHD7*$^{KO/KO}$ organoids highlighting the WT supporting cell cluster and the *CHD7*$^{KO/KO}$ otic-like cluster (dotted line). **b–c**, Bioinformatically isolated WT supporting cell cluster and *CHD7*$^{KO/KO}$ otic-like cluster plotted by cluster (**b**) or genotype (**c**). **d**, Volcano plot of differentially

expressed genes in *CHD7*$^{KO/KO}$ otic-like cells compared to WT supporting cells. **e**, Split-violin plots of key dysregulated genes as shown in (**d**). GO, gene ontology. **f**, Bubble plot of enriched gene sets from downregulated genes in d70 *CHD7*$^{KO/KO}$ otic-like cells compared to d70 WT supporting cells. For DE analysis in (**d**), the two-sided test DESeq2 was used, and adjustments were made by the Benjamini-Hochberg method for multiple comparisons. For GSEA using the one-sided iDEA platform shown in (**f**), adjustments of *p*-values were made by the Louis method.

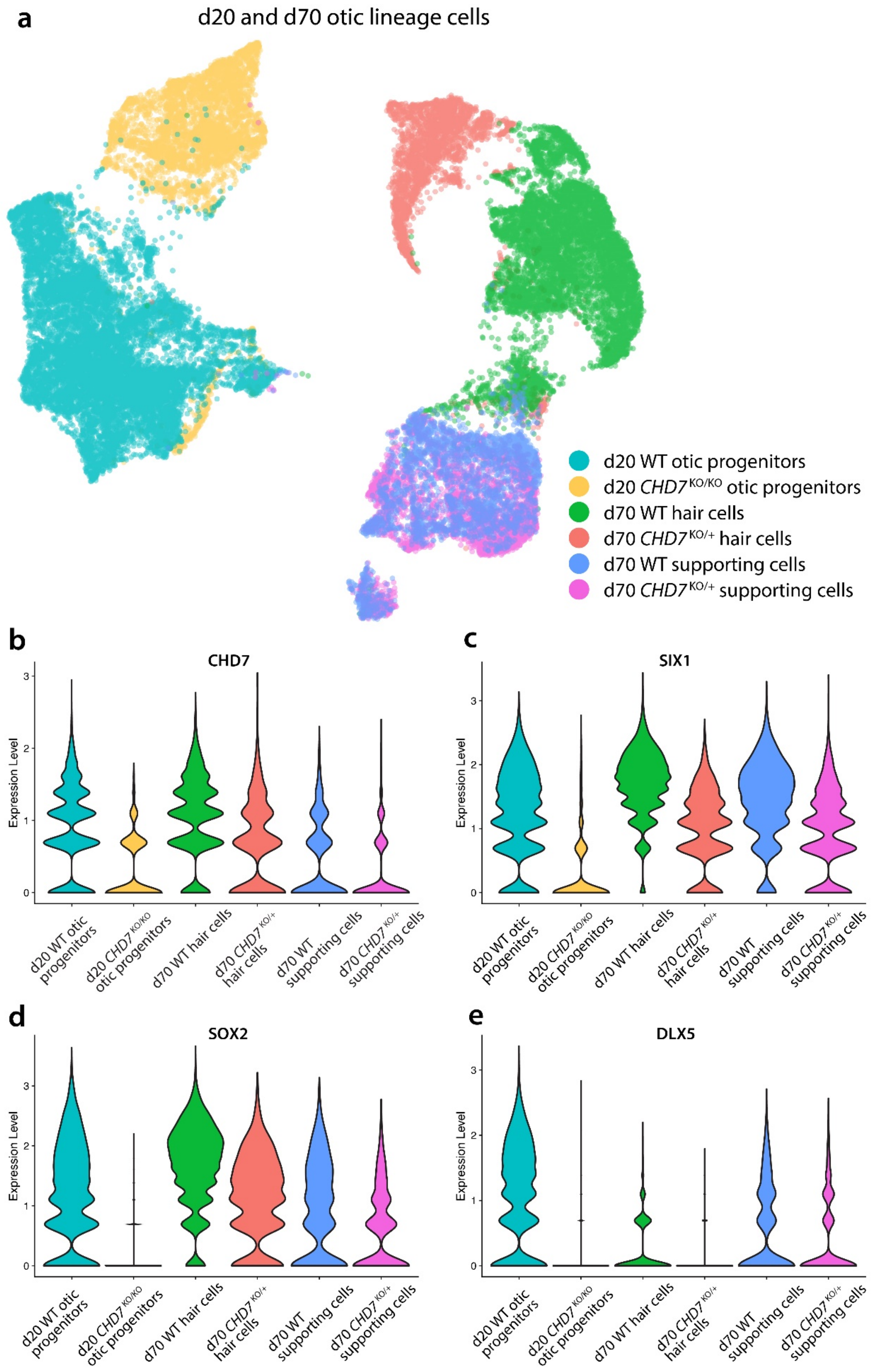

a
d20 and d70 otic lineage cells
d20 WT otic progenitors
d20 CHD7^KO/KO otic progenitors
d70 WT hair cells
d70 CHD7^KO/+ hair cells
d70 WT supporting cells
d70 CHD7^KO/+ supporting cells
b
CHD7
c
SIX1
d
SOX2
e
DLX5
Expression Level
0
1
2
3
d20 WT otic progenitors
d20 CHD7 KO/KO otic progenitors
d70 WT hair cells
d70 CHD7 KO/+ hair cells
d70 WT supporting cells
d70 CHD7 KO/+ supporting cells

Supplementary Figure 18. scRNA-seq analysis of merged d20 otic progenitor dataset (WT and *CHD7*$^{KO/KO}$) and d70 hair cell and supporting cell datasets (WT and *CHD7*$^{KO/+}$). **a**, UMAP plot of the merged datasets. **b–e**, Violin plots showing the expression levels of *CHD7*, *SIX1*, *SOX2*, and *DLX5* in d20 otic progenitors (WT and *CHD7*$^{KO/KO}$) and d70 hair cells and supporting cells (WT and *CHD7*$^{KO/+}$).

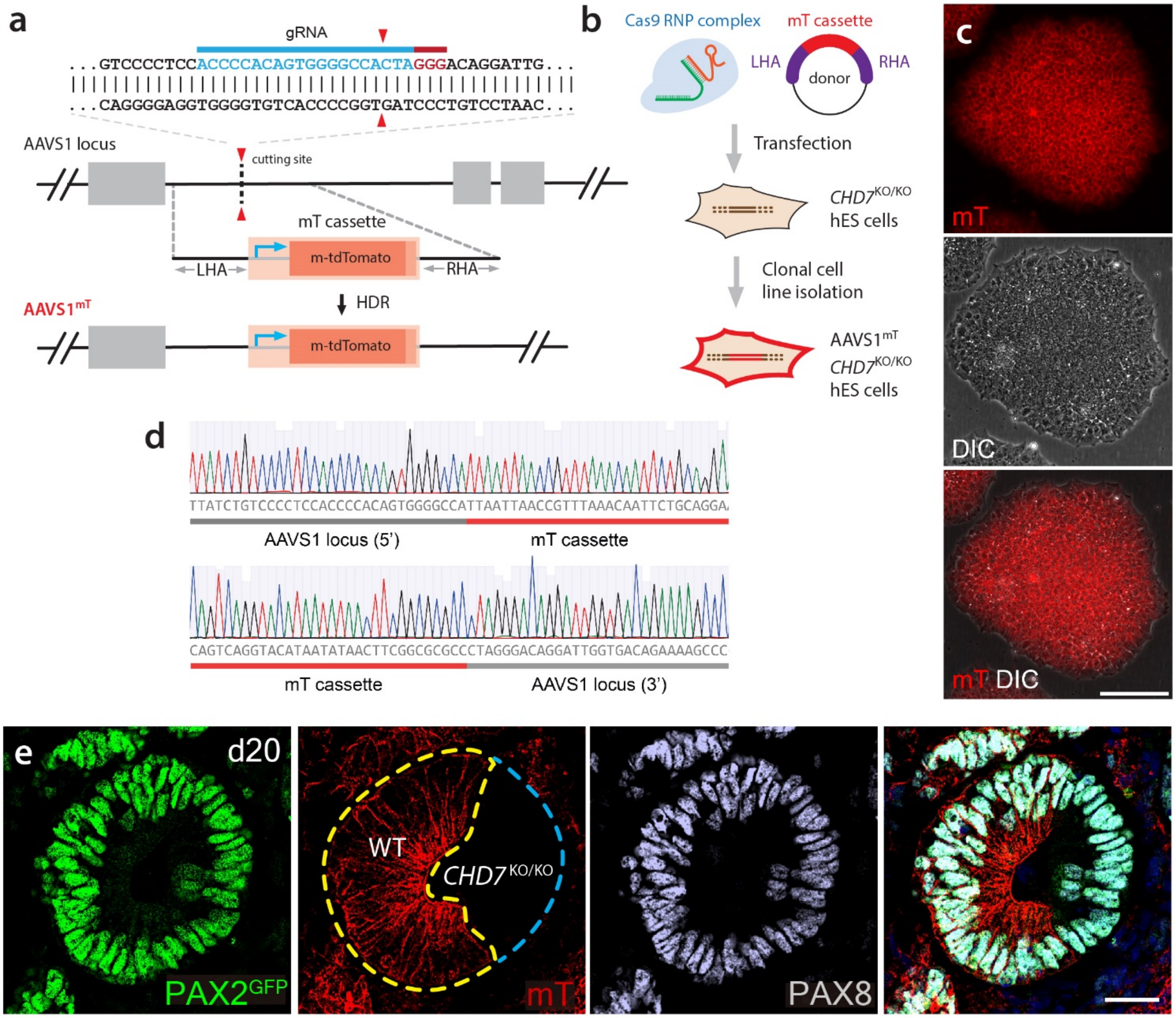

Supplementary Figure 19. Generation of AAVS1-pCA-mtdTomato (AAVS1^{mT}) knockin hESC line on the *CHD7*$^{KO/KO}$ genetic background. **a**, CRISPR genome engineering design of pCA-mtdTomato knockin to the AAVS1 locus. **b**, CRISPR knockin and clonal cell line isolation workflow. **c**, Membrane-bound tdTomato is expressed in AAVS1^{mT} hESCs. **d**, Sanger sequencing chromatograms of junction regions showing correct insertion of the pCA-mtdTomato cassette to the AAVS1 locus. **e**, In WT-*CHD7*$^{KO/KO}$ chimeric organoids, AAVS1^{mT} labeled the WT clones while the *CHD7*$^{KO/KO}$ were unlabeled. Scale bars, 50 µm (**c**), 25 µm (**e**).

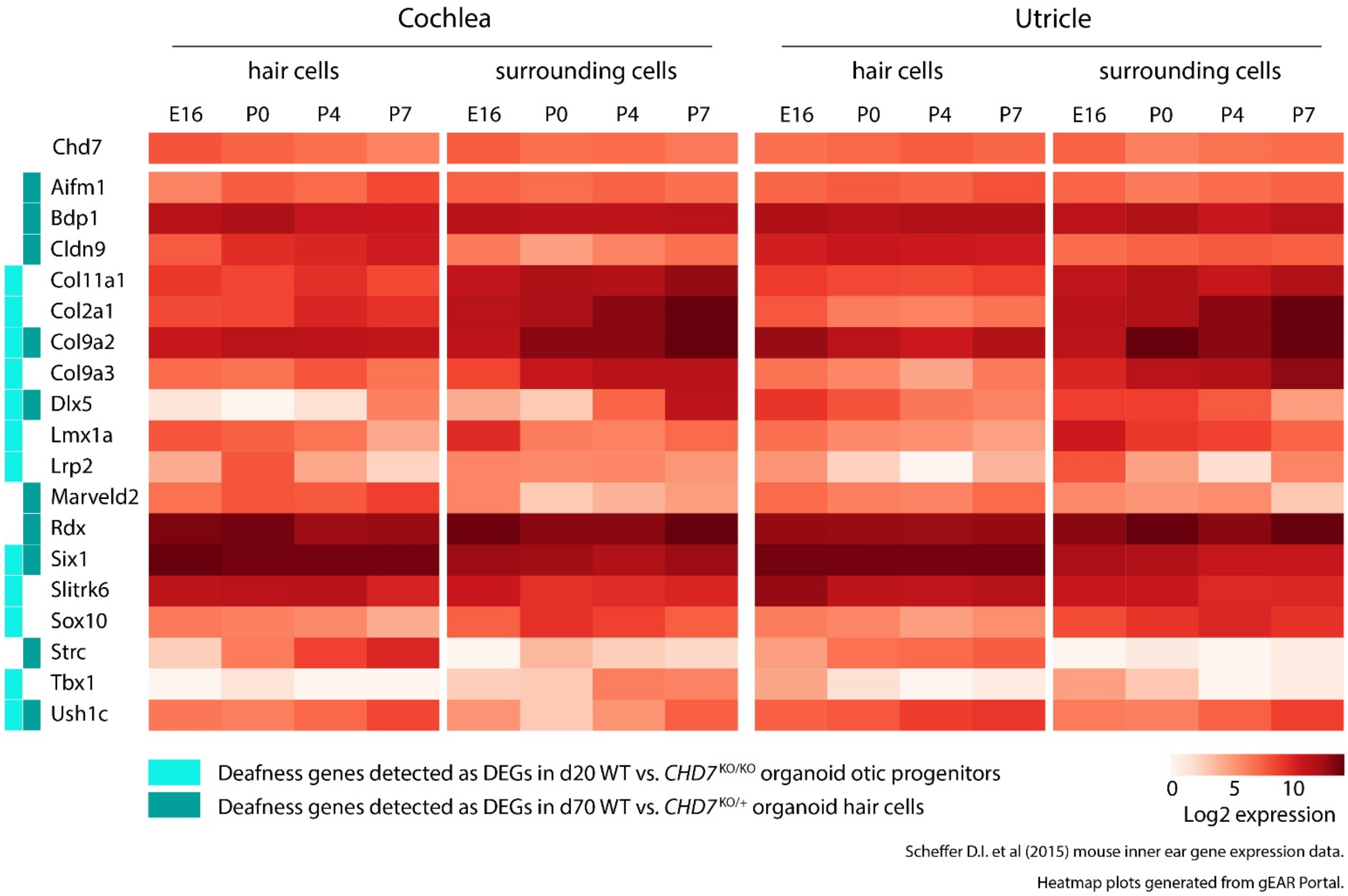

Supplementary Figure 20. Heatmap of *CHD7* and *CHD7* downstream deafness gene expression in cochlea and utricle hair cells and surrounding cells (including supporting cells) during mouse embryonic and postnatal development. RNA-seq gene expression data was obtained from Scheffer D.I. et al. (2015). Heatmap plot was generated from the gEAR portal (https://umgear.org/). The color bar to the bottom right shows the log2-normalized expression scale. The color bars to the left of each gene show whether they were detected as differentially expressed genes (DEGs) in d20 WT vs. *CHD7*$^{KO/KO}$ organoid otic progenitors or in d70 WT vs. *CHD7*$^{KO/+}$ hair cells.

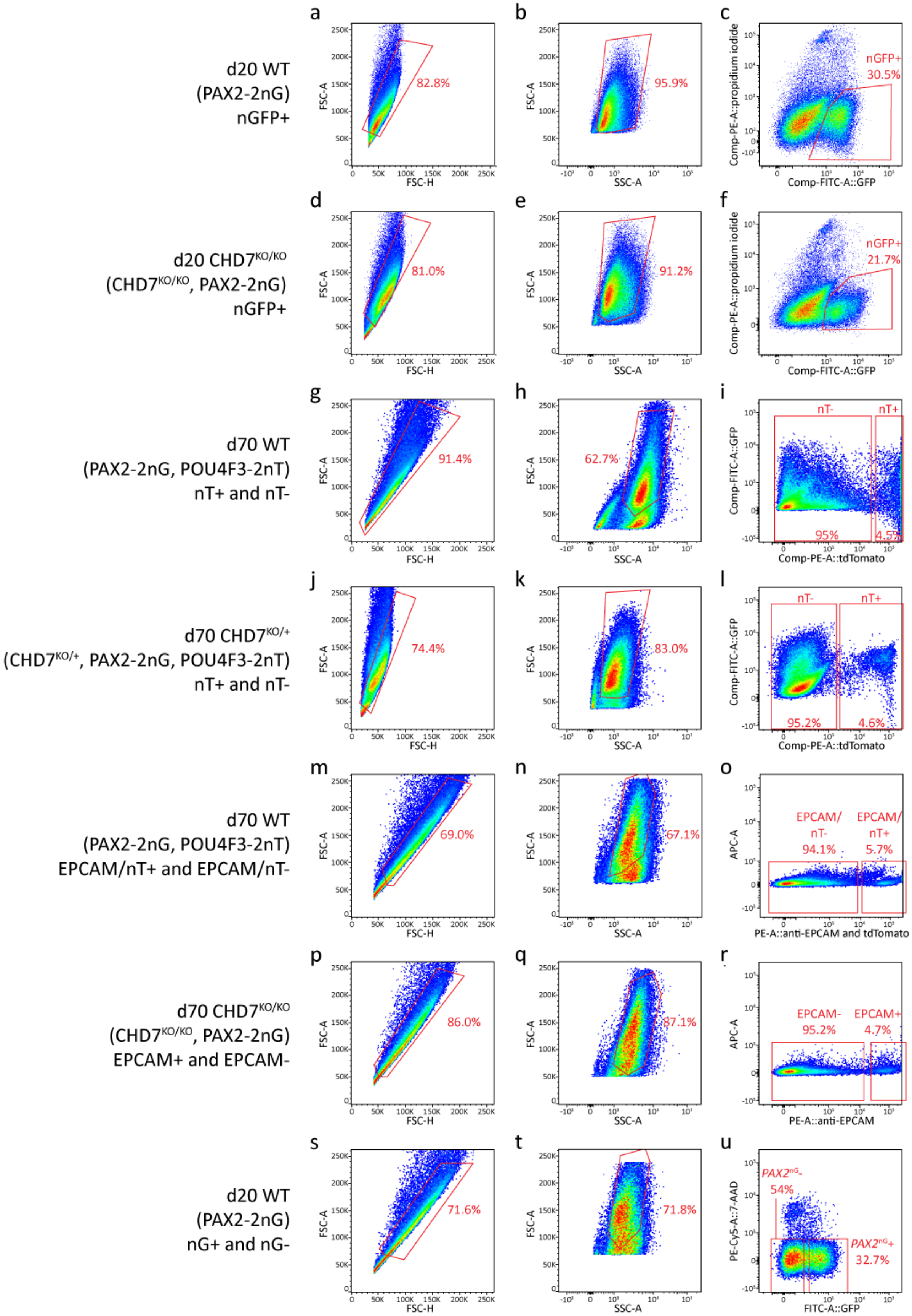

d20 WT
(PAX2-2nG)
nGFP+
d20 CHD7KO/KO
(CHD7KO/KO, PAX2-2nG)
nGFP+
d70 WT
(PAX2-2nG, POU4F3-2nT)
nT+ and nT-
d70 CHD7KO/+
(CHD7KO/+, PAX2-2nG, POU4F3-2nT)
nT+ and nT-
d70 WT
(PAX2-2nG, POU4F3-2nT)
EPCAM/nT+ and EPCAM/nT-
d70 CHD7KO/KO
(CHD7KO/KO, PAX2-2nG)
EPCAM+ and EPCAM-
d20 WT
(PAX2-2nG)
nG+ and nG-
a
82.8%
b
95.9%
c
nGFP+
30.5%
d
81.0%
e
91.2%
f
nGFP+
21.7%
g
91.4%
h
62.7%
i
nT-
nT+
95%
4.5%
j
74.4%
k
83.0%
l
nT-
nT+
95.2%
4.6%
m
69.0%
n
67.1%
o
EPCAM/
nT-
94.1%
EPCAM/
nT+
5.7%
p
86.0%
q
87.1%
r
EPCAM-
95.2%
EPCAM+
4.7%
s
71.6%
t
71.8%
u
PAX2nG-
54%
PAX2nG+
32.7%
FSC-A
FSC-H
SSC-A
Comp-PE-A::propidium iodide
Comp-FITC-A::GFP
Comp-PE-A::tdTomato
APC-A
PE-A::anti-EPCAM and tdTomato
PE-A::anti-EPCAM
PE-Cy5-A::7-AAD
FITC-A::GFP

Supplementary Figure 21. FACS sequential gating strategies. **a**–**f**, FACS sequential gating strategies of *PAX2*-2a-nGFP^{+} cell isolation from d20 WT and d20 *CHD7*$^{KO/KO}$ organoids as shown in Supplementary Figure 7a middle and right panels, respectively. **g**–**l**, FACS sequential gating strategies of *POU4F3*-2a-ntdTomato^{+} and *POU4F3*-2a-ntdTomato^{-} cell isolation from d70 WT and d70 *CHD7*$^{KO/+}$ organoids as shown in Supplementary Figure 11a middle and right panels, respectively. **m**–**r**, FACS sequential gating strategies of EPCAM^{+} and EPCAM^{-} cell isolation from d70 WT and d70 *CHD7*$^{KO/KO}$ organoids as shown in Supplementary Figure 14a middle and right panels, respectively. The *POU4F3*^{nT}+ hair cells in d70 WT organoids endogenously emit tdTomato fluorescence. These hair cells were also stained positive for PE-conjugated EPCAM, making them double-positive for these two red fluorescence signals. **s**–**u**, FACS sequential gating strategies of *PAX2*-2a-nGFP^{+} and *PAX2*-2a-nGFP^{-} cell isolation from d20 WT organoids as shown in Supplementary Figure 8a right panel.

Supplementary Table 1. Antibodies used in this study.

antibody	host	mAb clone	supplier	cat. No.	dilution	Validation reference
ANXA4	goat	N/A	R&D Systems	AF4146	1:100	[1]
CDH1	mouse	36	BD Biosciences	610181	1:200	[2]
CHD7	rabbit	D3F5	Cell Signaling	6505S	1:400 WB	[3]
CHD7*	sheep	N/A	R&D Systems	AF7350	1:50 IHC, 1:400 WB	[4]
CHD7	rabbit	N/A	Abcam	ab31824	1:400 WB	[5]
COL9A2	rabbit	N/A	Sigma	HPA056316	1:100	[6]
DLX5	rabbit	N/A	Novus Biologicals	NBP1-19547	1:100	[7]
EPCAM	mouse	9C4	BioLegend	324202	1:200	[8]
EPCAM (conjugated to PE)	mouse	9C4	BioLegend	324206	1:200	[9]
FBXO2	mouse	E-9	Santa Cruz	sc-398111	1:25	[10]
Flag	mouse	M2	Sigma-Aldrich	F1804	1:100 IHC, 1:400 WB	[11]
GAPDH (conjugated to HRP)	mouse	GA1R	Invitrogen	MA515738HRP	1:2000 WB	[12]
GFP	mouse	3E6	Thermo Fisher	A-11120	1:100	[13]
HOXB9	rabbit	E7P5O	Cell Signaling	27967S	1:100	[14]
MYO7A	rabbit	N/A	Proteus	256790	1:100	[10]
MYO7A	mouse	C-5	Santa Cruz	sc-74516	1:20	[15]
NEFL	rabbit	N/A	Millipore	Ab9568	1:200	[10]
OCT4 (conjugated to AF488)	rat	EM92	eBioscience	53584182	1:100	[10]
PAX8	rabbit	N/A	Abcam	AB97477	1:100	[10]
PCP4	rabbit	N/A	Santa Cruz	sc-74816	1:400	[1]
phalloidin (conjugated to AF488)	N/A	N/A	Thermo Fisher	A12379	1:100	[10]
POU4F3	mouse	QQ8	Santa Cruz	sc-81980	1:25	[10]
S100B	rabbit	EP1576Y	Abcam	Ab52642	1:100	[16]
SIX1	rabbit	D4A8K	Cell Signaling	12891	1:100	[17]
SOX10	mouse	20B7	eBioscience	14-5923-82	1:50	[18]
SOX2	mouse	O30-678	BD Pharmingen	561469	1:200	[10]
SOX2	rabbit	N/A	Millipore Sigma	AB5603	1:100	[19]
SPARCL1	goat	N/A	R&D Systems	AF2728	1:100	[1]
SSEA4 (conjugated to AF594)	mouse	MC-813-70	BioLegend	330414	1:200	[10]
TFAP2A	mouse	3B5	DSHB	3B5	1:5	[10]

Abbreviations: mAb, monoclonal antibody. HRP, horseradish peroxidase. AF488, Alexa Fluor 488. AF594, Alexa Fluor 594. IHC, immunohistochemistry. PE, phycoerythrin. WB, western blot.

* Unless otherwise noted, all anti-CHD7 IHC and WB experiments in this study used this antibody.

Supplementary Table 2. Known vestibular dysfunctions of deafness genes dysregulated in *CHD7* mutant organoids.

Gene	Dysregulation in *CHD7* mutant organoids		OtoSCOPE deafness gene?	Known vestibular dysfunctions or defects?
	in d20 *CHD7*$^{KO/KO}$ otic progenitors	in d70 *CHD7*$^{KO/+}$ hair cells		
AIFM1		Down-regulation	Yes	Yes[20]
ANKH	Down-regulation		Yes	Unknown
BDP1		Down-regulation	Yes	Unknown
CLDN9		Down-regulation	Yes	Unknown
COL11A1	Down-regulation		Yes	Yes[21]
COL2A1	Down-regulation		Yes	Yes[22]
COL9A2	Down-regulation	Down-regulation	Yes	Unknown
COL9A3	Down-regulation		Yes	Unknown
DLX5	Down-regulation	Down-regulation	Yes	Yes[23]
LMX1A	Down-regulation		Yes	Yes[24]
LRP2	Down-regulation		Yes	Yes[25]
MARVELD2		Down-regulation	Yes	Unknown
MT-ND1		Down-regulation	Yes	Yes[26]
OTOGL	Down-regulation	Up-regulation	Yes	Yes[27]
RDX		Down-regulation	Yes	Unknown
SIX1	Down-regulation	Down-regulation	Yes	Yes[28]
SLITRK6	Down-regulation		Yes	Yes[29]
SOX10	Down-regulation		Yes	Yes[30]
STRC		Up-regulation	Yes	Yes[31]
TBX1	Down-regulation		Yes	Yes[32]
USH1C	Down-regulation	Down-regulation	Yes	Yes[33]

Supplementary References

1. Burns JC, Kelly MC, Hoa M, Morell RJ, Kelley MW. Single-cell RNA-Seq resolves cellular complexity in sensory organs from the neonatal inner ear. *Nat Commun* **6**, 8557 (2015).

2. Koehler KR, Mikosz AM, Molosh AI, Patel D, Hashino E. Generation of inner ear sensory epithelia from pluripotent stem cells in 3D culture. *Nature* **500**, 217-221 (2013).

3. He D*, et al.* Chd7 cooperates with Sox10 and regulates the onset of CNS myelination and remyelination. *Nat Neurosci* **19**, 678-689 (2016).

4. Yamamoto T*, et al.* Differentiation potential of Pluripotent Stem Cells correlates to the level of CHD7. *Sci Rep* **8**, 241 (2018).

5. Engelen E*, et al.* Sox2 cooperates with Chd7 to regulate genes that are mutated in human syndromes. *Nat Genet* **43**, 607-611 (2011).

6. Hartman BH, Durruthy-Durruthy R, Laske RD, Losorelli S, Heller S. Identification and characterization of mouse otic sensory lineage genes. *Front Cell Neurosci* **9**, 79 (2015).

7. Bellessort B*, et al.* Dlx5 and Dlx6 control uterine adenogenesis during post-natal maturation: possible consequences for endometriosis. *Hum Mol Genet* **25**, 97-108 (2016).

8. Wagner J*, et al.* A Single-Cell Atlas of the Tumor and Immune Ecosystem of Human Breast Cancer. *Cell* **177**, 1330-1345 e1318 (2019).

9. Busslinger GA, Weusten BLA, Bogte A, Begthel H, Brosens LAA, Clevers H. Human gastrointestinal epithelia of the esophagus, stomach, and duodenum resolved at single-cell resolution. *Cell Rep* **34**, 108819 (2021).

10. Koehler KR*, et al.* Generation of inner ear organoids containing functional hair cells from human pluripotent stem cells. *Nat Biotechnol* **35**, 583-589 (2017).

11. Chu J*, et al.* Rocaglates Induce Gain-of-Function Alterations to eIF4A and eIF4F. *Cell Rep* **30**, 2481-2488 e2485 (2020).

12. Wei LY*, et al.* Overexpression of ABCG2 confers resistance to pevonedistat, an NAE inhibitor. *Exp Cell Res* **388**, 111858 (2020).

13. Shao L*, et al.* Generation of iPS cells using defined factors linked via the self-cleaving 2A sequences in a single open reading frame. *Cell Res* **19**, 296-306 (2009).

14. Szatanek T. HOXB9 (E7P5O) Rabbit mAb #27967.). Revision 1 edn (7/9/2020).

15. Grati M, Kachar B. Myosin VIIa and sans localization at stereocilia upper tip-link density implicates these Usher syndrome proteins in mechanotransduction. *Proc Natl Acad Sci U S A* **108**, 11476-11481 (2011).

16. Duan R*, et al.* Tumor Microenvironment Status Predicts the Efficacy of Postoperative Chemotherapy or Radiochemotherapy in Resected Gastric Cancer. *Front Immunol* **11**, 609337 (2020).

17. Xu J, Li J, Ramakrishnan A, Yan H, Shen L, Xu PX. Six1 and Six2 of the Sine Oculis Homeobox Subfamily are Not Functionally Interchangeable in Mouse Nephron Formation. *Front Cell Dev Biol* **10**, 815249 (2022).

18. Ferletta M, Uhrbom L, Olofsson T, Ponten F, Westermark B. Sox10 has a broad expression pattern in gliomas and enhances platelet-derived growth factor-B--induced gliomagenesis. *Mol Cancer Res* **5**, 891-897 (2007).

19. Brown RM, 2nd, Nelson JC, Zhang H, Kiernan AE, Groves AK. Notch-mediated lateral induction is necessary to maintain vestibular prosensory identity during inner ear development. *Dev Biol* **462**, 74-84 (2020).

20. Wang H*, et al.* High Frequency of AIFM1 Variants and Phenotype Progression of Auditory Neuropathy in a Chinese Population. *Neural Plast* **2020**, 5625768 (2020).

21. Griffith AJ, Gebarski SS, Shepard NT, Kileny PR. Audiovestibular phenotype associated with a COL11A1 mutation in Marshall syndrome. *Arch Otolaryngol Head Neck Surg* **126**, 891-894 (2000).

22. Donahue LR*, et al.* A missense mutation in the mouse Col2a1 gene causes spondyloepiphyseal dysplasia congenita, hearing loss, and retinoschisis. *J Bone Miner Res* **18**, 1612-1621 (2003).

23. Merlo GR*, et al.* The Dlx5 homeobox gene is essential for vestibular morphogenesis in the mouse embryo through a BMP4-mediated pathway. *Dev Biol* **248**, 157-169 (2002).

24. Wesdorp M*, et al.* Heterozygous missense variants of LMX1A lead to nonsyndromic hearing impairment and vestibular dysfunction. *Hum Genet* **137**, 389-400 (2018).

25. Kantarci S*, et al.* Mutations in LRP2, which encodes the multiligand receptor megalin, cause Donnai-Barrow and facio-oculo-acoustico-renal syndromes. *Nat Genet* **39**, 957-959 (2007).

26. La Morgia C*, et al.* Association of the mtDNA m.4171C>A/MT-ND1 mutation with both optic neuropathy and bilateral brainstem lesions. *BMC Neurol* **14**, 116 (2014).

27. Skuladottir AT*, et al.* A genome-wide meta-analysis uncovers six sequence variants conferring risk of vertigo. *Commun Biol* **4**, 1148 (2021).

28. Bosman EA, Quint E, Fuchs H, Hrabe de Angelis M, Steel KP. Catweasel mice: a novel role for Six1 in sensory patch development and a model for branchio-oto-renal syndrome. *Dev Biol* **328**, 285-296 (2009).

29. Matsumoto Y*, et al.* Impaired auditory-vestibular functions and behavioral abnormalities of Slitrk6-deficient mice. *PLoS One* **6**, e16497 (2011).

30. Qi JC*, et al.* Sox10 Gene Is Required for the Survival of Saccular and Utricular Hair Cells in a Porcine Model. *Mol Neurobiol* **59**, 3323-3335 (2022).

31. Frykholm C, Klar J, Tomanovic T, Ameur A, Dahl N. Stereocilin gene variants associated with episodic vertigo: expansion of the DFNB16 phenotype. *Eur J Hum Genet* **26**, 1871-1874 (2018).

32. Tian C, Johnson KR. TBX1 is required for normal stria vascularis and semicircular canal development. *Dev Biol* **457**, 91-103 (2020).

33. Pan B*, et al.* Gene therapy restores auditory and vestibular function in a mouse model of Usher syndrome type 1c. *Nat Biotechnol* **35**, 264-272 (2017).

Uncropped gels and blots

Supplementary Figure 1a.

Top left panel

Anti-CHD7 (R&D systems #AF7350)

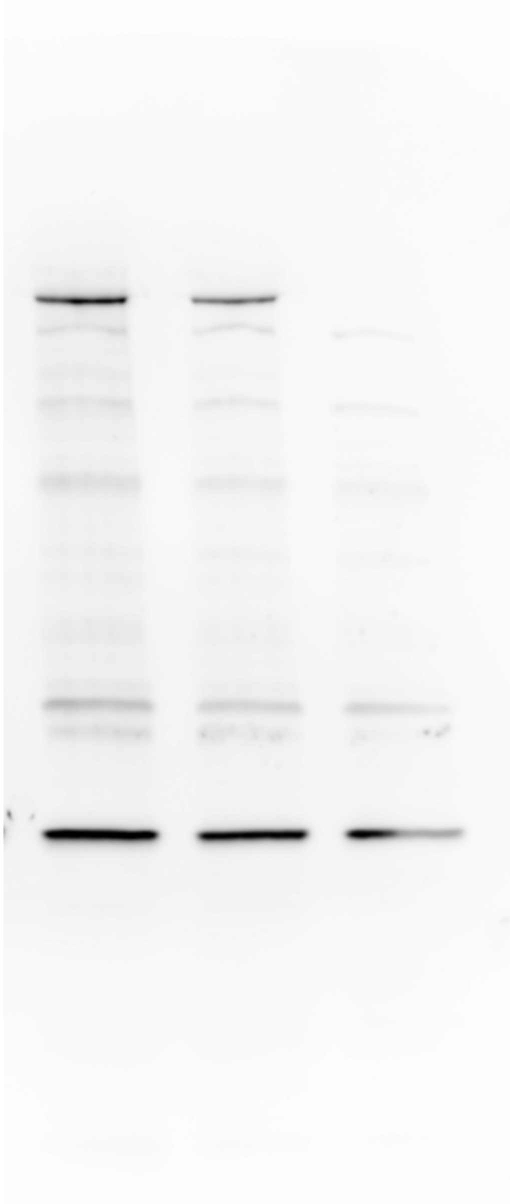

Supplementary Figure 1a.

Top middle panel

Anti-CHD7 (Cell Signaling #6505S)

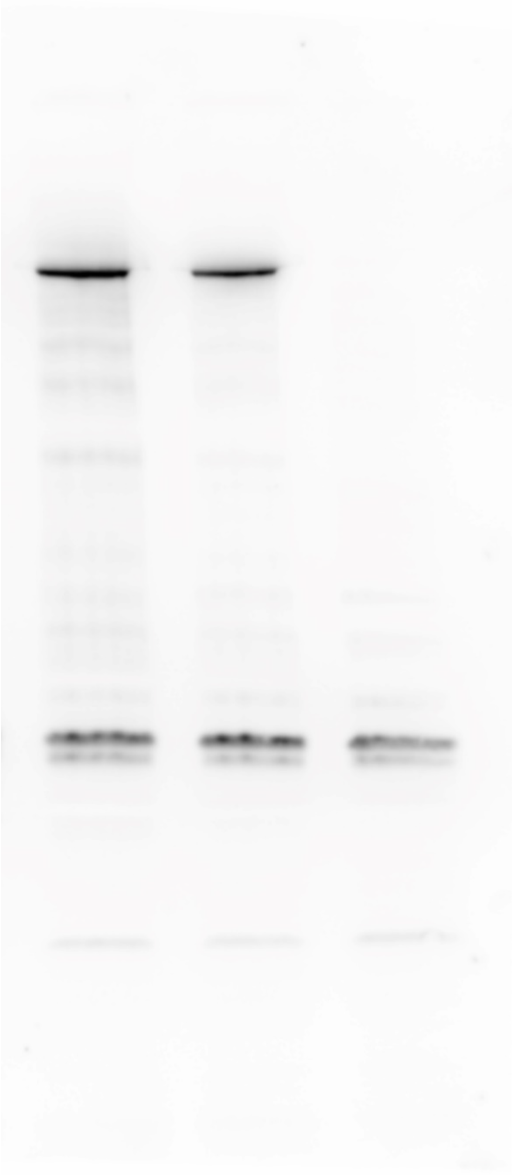

Supplementary Figure 1a.

Top right panel

Anti-CHD7 (Abcam #ab31824)

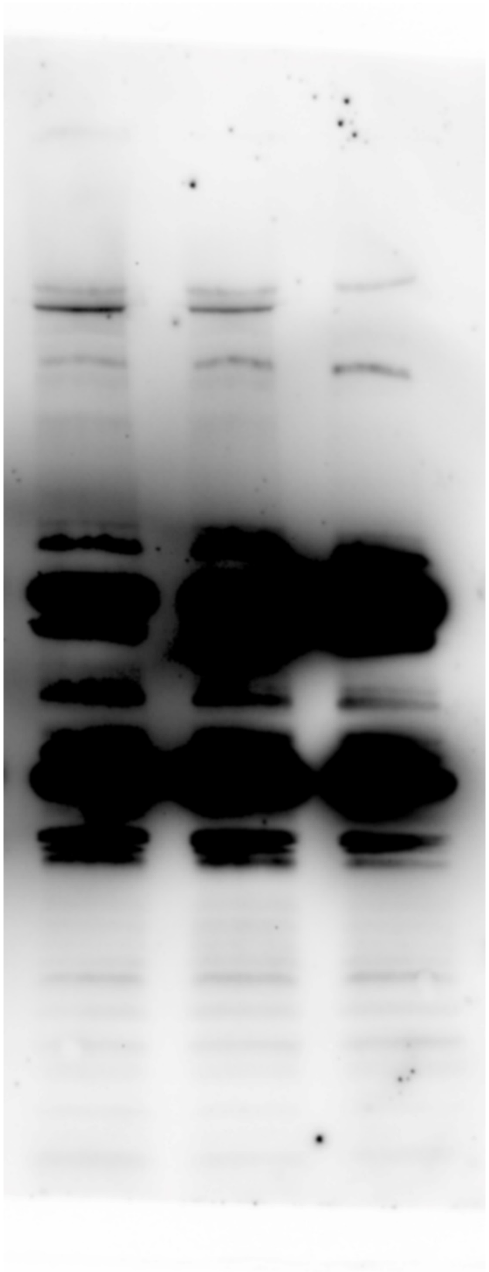

Supplementary Figure 1a.

Bottom panel

Anti-GAPDH

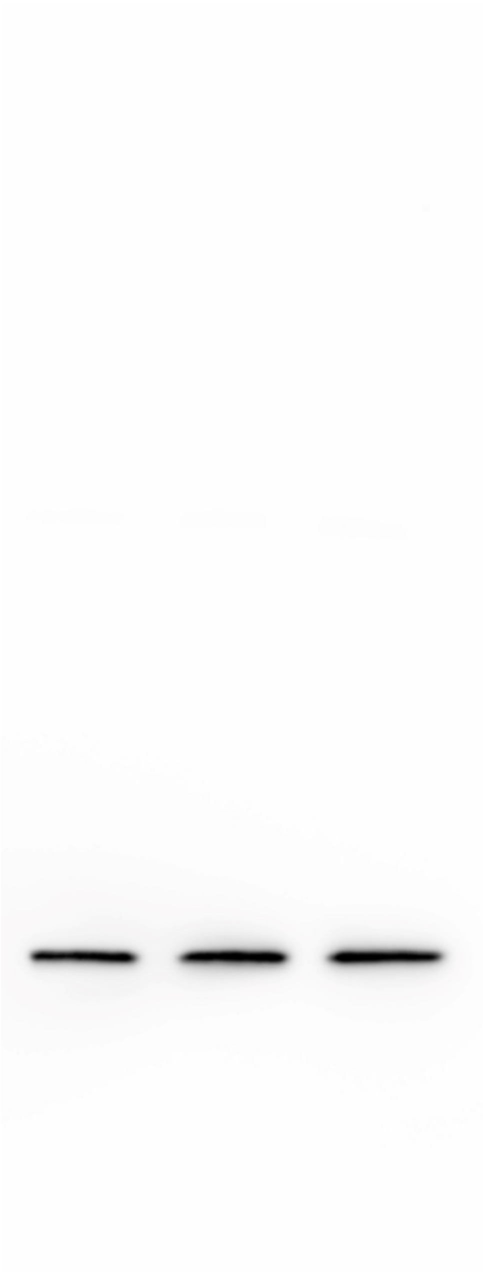

Supplementary Figure 2b.

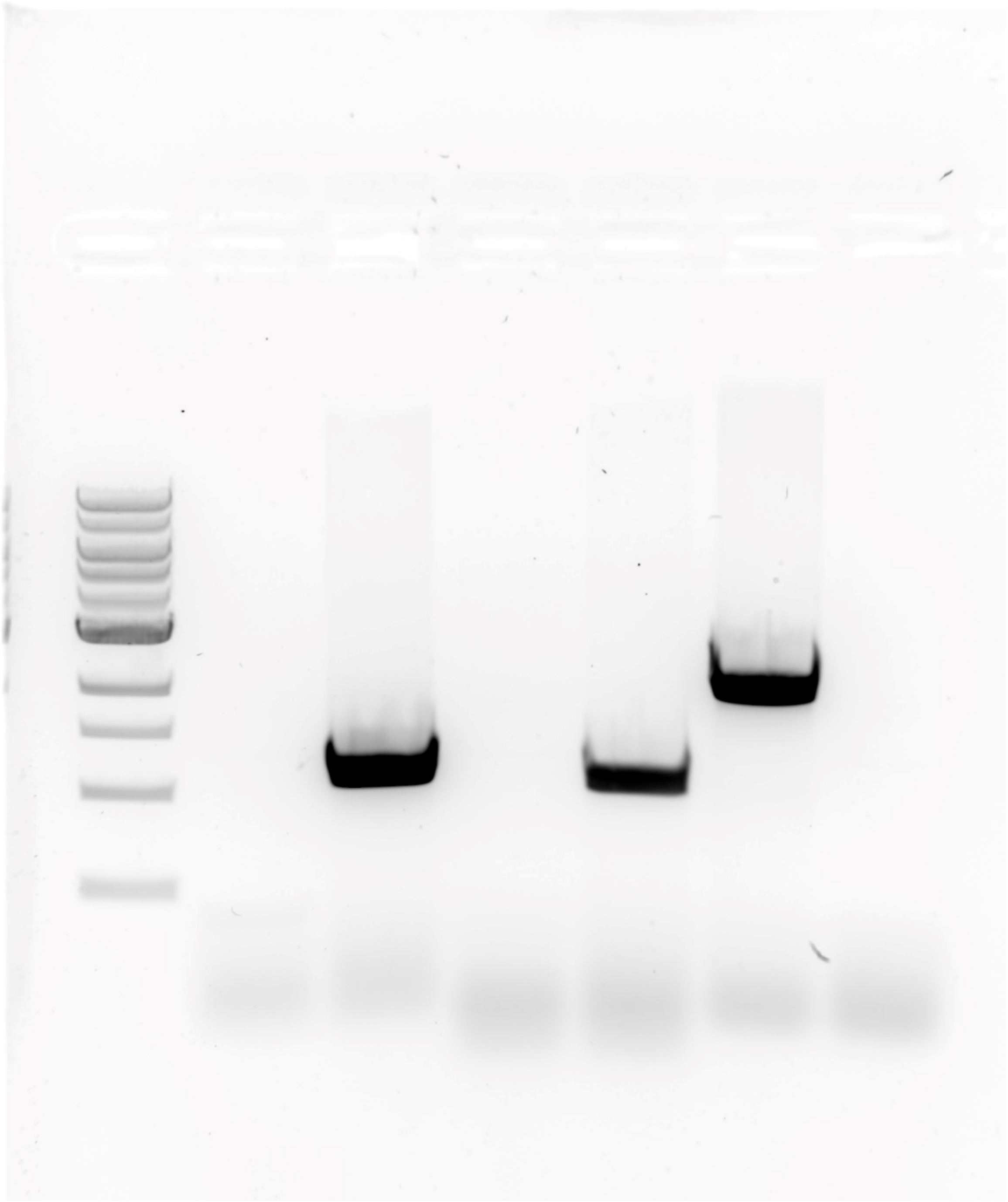

Supplementary Figure 3b.

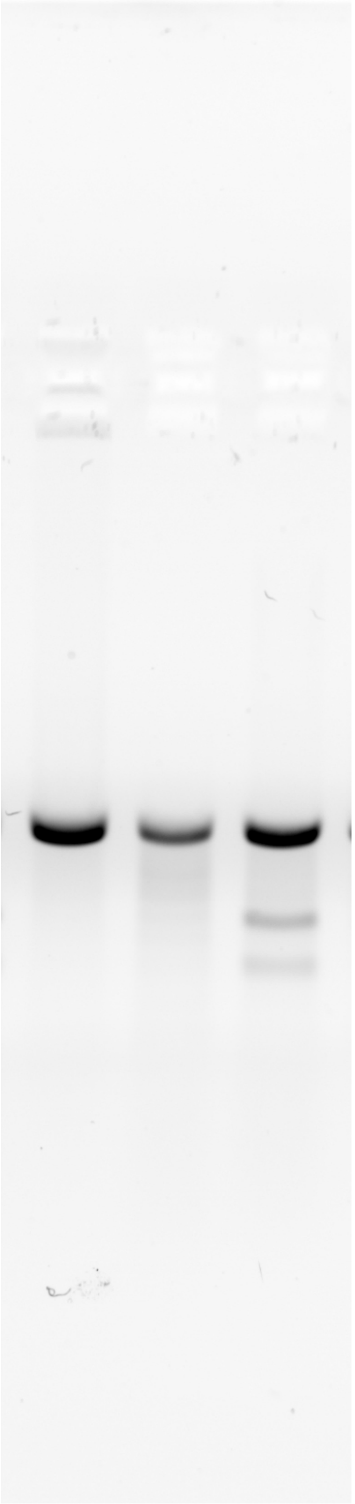

Supplementary Figure 3c.

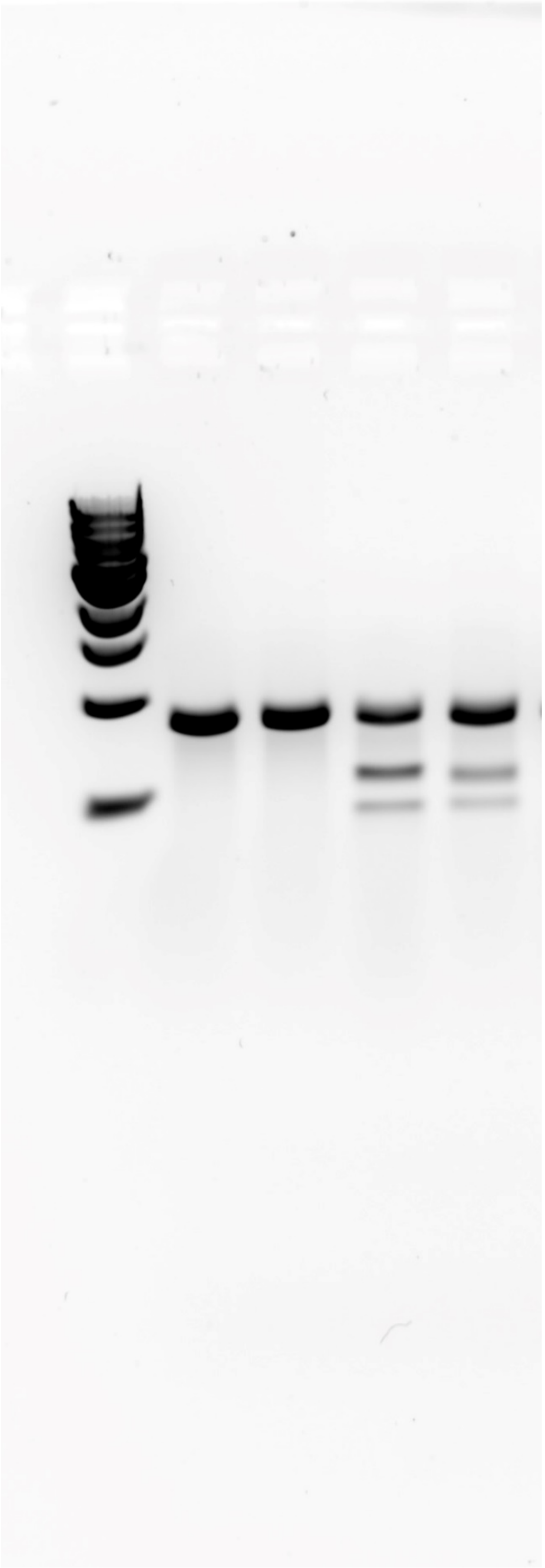

Supplementary Figure 5b.

Top panel

Anti-CHD7

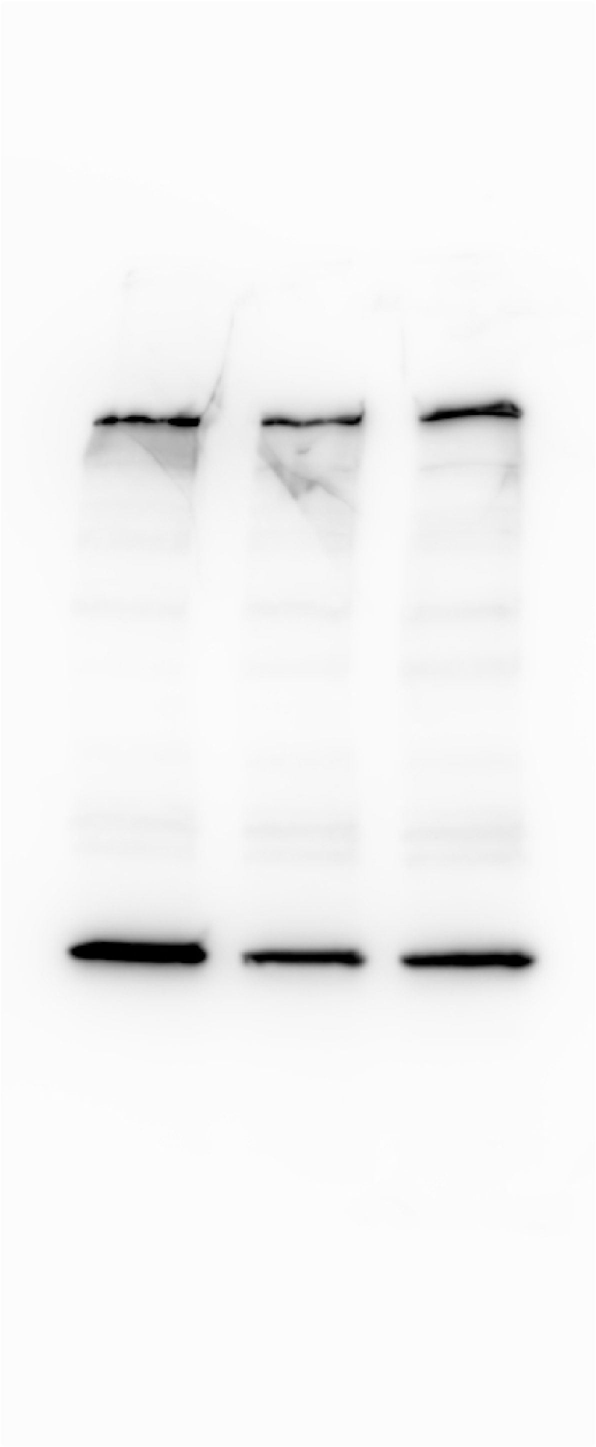

Supplementary Figure 5b.

Bottom panel

Anti-GAPDH

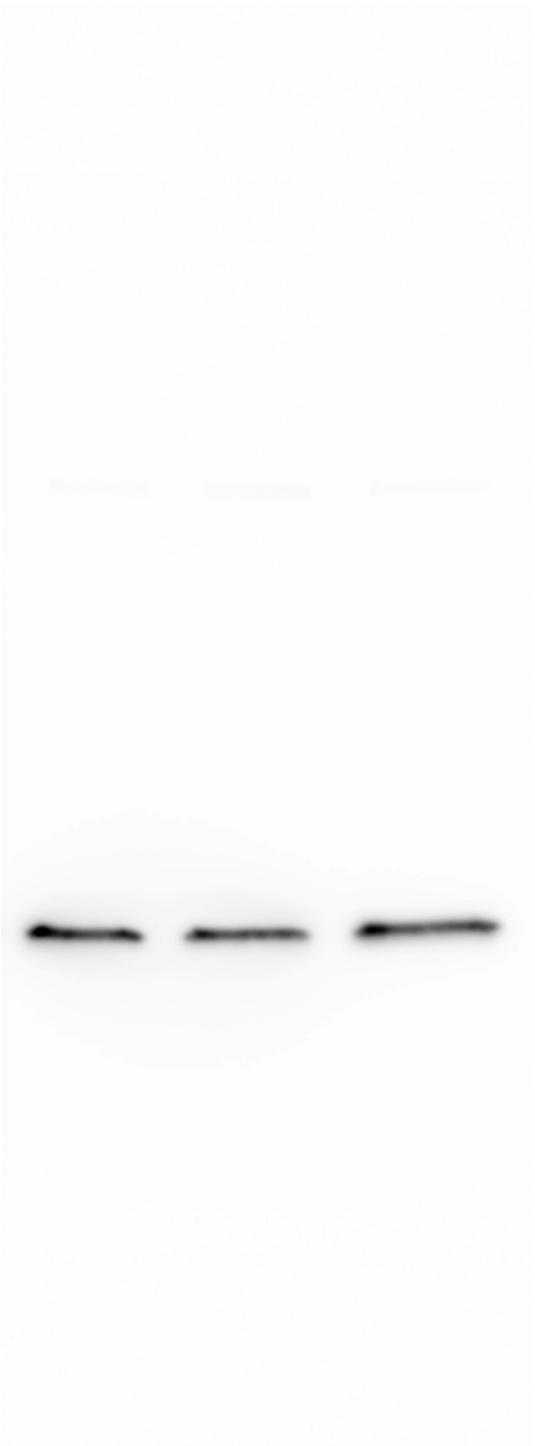

Supplementary Figure 10b.

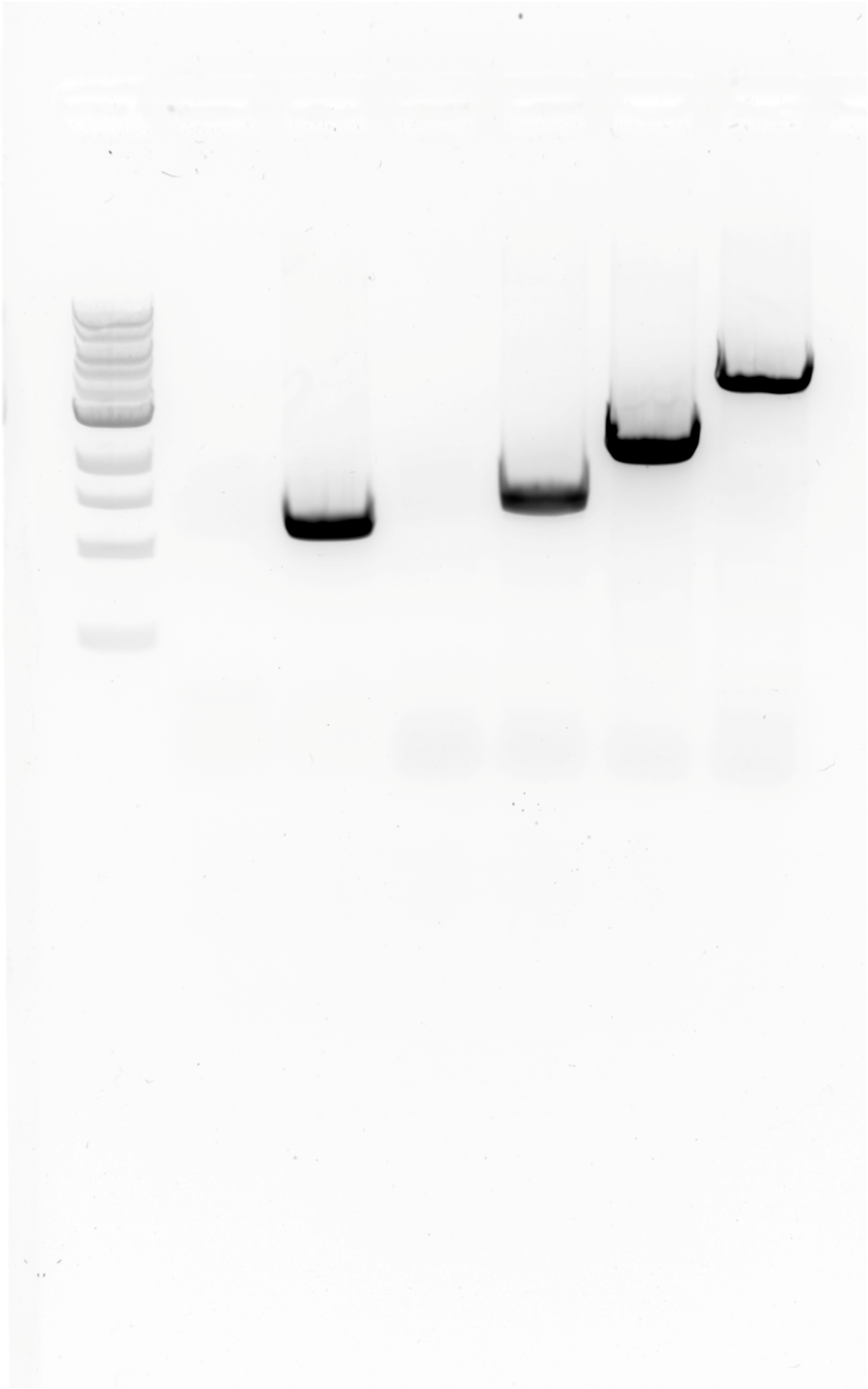

Supplement: Supplementary file 1 — Supplementary Information [file 41467_2022_34759_MOESM1_ESM.pdf]
