# Supplementary material for: CHD7 regulates otic lineage specification and hair cell differentiation in human inner ear organoids
Source: Nat Commun. 2022 Nov 17;13:7053. doi: 10.1038/s41467-022-34759-8 (PMC9672366; doi:10.1038/s41467-022-34759-8)
Supplement: Supplementary file 3 — Reporting Summary [file 41467_2022_34759_MOESM3_ESM.pdf]

nature portfolio

Corresponding author(s): Eri Hashino
Last updated by author(s): 10/25/2022

Reporting Summary

Nature Portfolio wishes to improve the reproducibility of the work that we publish. This form provides structure for consistency and transparency in reporting. For further information on Nature Portfolio policies, see our Editorial Policies and the Editorial Policy Checklist.

Statistics

For all statistical analyses, confirm that the following items are present in the figure legend, table legend, main text, or Methods section.

n/a	Confirmed	
☐	☒	The exact sample size (*n*) for each experimental group/condition, given as a discrete number and unit of measurement
☐	☒	A statement on whether measurements were taken from distinct samples or whether the same sample was measured repeatedly
☐	☒	The statistical test(s) used AND whether they are one- or two-sided<br>*Only common tests should be described solely by name; describe more complex techniques in the Methods section.*
☒	☐	A description of all covariates tested
☒	☐	A description of any assumptions or corrections, such as tests of normality and adjustment for multiple comparisons
☐	☒	A full description of the statistical parameters including central tendency (e.g. means) or other basic estimates (e.g. regression coefficient) AND variation (e.g. standard deviation) or associated estimates of uncertainty (e.g. confidence intervals)
☐	☒	For null hypothesis testing, the test statistic (e.g. *F*, *t*, *r*) with confidence intervals, effect sizes, degrees of freedom and *P* value noted<br>*Give P values as exact values whenever suitable.*
☒	☐	For Bayesian analysis, information on the choice of priors and Markov chain Monte Carlo settings
☒	☐	For hierarchical and complex designs, identification of the appropriate level for tests and full reporting of outcomes
☒	☐	Estimates of effect sizes (e.g. Cohen's *d*, Pearson's *r*), indicating how they were calculated

Our web collection on statistics for biologists contains articles on many of the points above.

Software and code

Policy information about availability of computer code

Data collection	The Fiji/ImageJ (version 1.53f51) software was used to measure fluorescence intensity data.
Data analysis	For scRNA-seq analyses, we used bcl2fastQ v2.20, Cell Ranger v4.0.0, Seurat v4.0.3, DESeq2 v1.32.0, EnhancedVolcano v1.10.0, ZingeR v0.1.0, and iDEA v1.0.1 platforms. For statistical analyses, we used GraphPad Prism 9. For image analyses, we used Fiji/ImageJ v1.53f51, Nikon NIS Elements Advanced Research v5.21.02, and Leica LAS X v3.7.4.23463 software. For CRISPR analysis, we used EditR v1.0.0. For flow cytometry, we used FlowJo v10.8.1.<br>Scripts used for scRNA-seq analysis are available at https://github.com/HashinoLab/Nie_et_al_CHD7 with DOI: 10.5281/zenodo.7139816.

For manuscripts utilizing custom algorithms or software that are central to the research but not yet described in published literature, software must be made available to editors and reviewers. We strongly encourage code deposition in a community repository (e.g. GitHub). See the Nature Portfolio guidelines for submitting code & software for further information.

Data

Policy information about availability of data

All manuscripts must include a data availability statement. This statement should provide the following information, where applicable:

- Accession codes, unique identifiers, or web links for publicly available datasets
- A description of any restrictions on data availability
- For clinical datasets or third party data, please ensure that the statement adheres to our policy

The scRNA-seq data generated in this study have been uploaded to the Gene Expression Omnibus with accession code GSE208585 (https://www.ncbi.nlm.nih.gov/geo/query/acc.cgi?acc=GSE208585). This study used the GRCh38/hg38 human reference genome dataset (https://www.ncbi.nlm.nih.gov/assembly/GCF_000001405.26/). Source data generated in this study are provided with this paper.

Human research participants

Policy information about studies involving human research participants and Sex and Gender in Research.

Reporting on sex and gender	N/A
Population characteristics	N/A
Recruitment	N/A
Ethics oversight	N/A

Note that full information on the approval of the study protocol must also be provided in the manuscript.

Field-specific reporting

Please select the one below that is the best fit for your research. If you are not sure, read the appropriate sections before making your selection.

☒ Life sciences ☐ Behavioural & social sciences ☐ Ecological, evolutionary & environmental sciences

For a reference copy of the document with all sections, see nature.com/documents/nr-reporting-summary-flat.pdf

Life sciences study design

All studies must disclose on these points even when the disclosure is negative.

Sample size	No power analysis was performed to determine the sample size. All immunostaining of organoids shown in this article are representative of a minimum of three aggregates from a minimum of three independent experiments. ~30 d20 aggregates and ~40-50 d70 aggregates were used for scRNA-seq experiments. These numbers were chosen based on previous studies in the organoid literature and based on our pilot runs for testing number of target cells (e.g. d20 PAX2+ otic progenitors and d70 POU4F3+ hair cells) that can be isolated from organoids. We target 10,000 cells per sample, which is roughly the maximum cell number that can be loaded per scRNA-seq reaction. Typically, we can isolate 1.5 - 2 million PAX2-2nG+ otic progenitor cells from the ~30 d20 organoids, which is more than enough for the targeted 10,000 cells. With ~40-50 d70 aggregates, we can typically isolate ~12,000 - 15,000 POU4F3-2nT+ hair cells after FACS sorting.
Data exclusions	For single-cell RNA-sequencing data, we follow the standard scRNA-seq data analysis workflow and filter cells with very low and high nFeature_RNA, cells with very high nCount_RNA, and cells with high mitochonrial expression percentage based on nFeature_RNA, nCount_RNA, and mt.per violin plot analysis results. No data were excluded for other experiments.
Replication	The images are representative of specimens obtained from at least three separate experiments. For immunohistochemical analysis of aggregates, we typically sectioned 6-20 aggregates from each condition in each experiment. Not all organoid in vitro differentiation experiments were successful and there were failed runs with very few otic vesicles and hair cells generated. But for the successful differentiation runs, all attempts at replication were successful.
Randomization	Not all aggregates with the same genotype from the same batch of organoid culture can produce otic vesicles or hair cells. As such, the samples were not randomly collected for immunostaining or scRNA-seq experiments. Prior to harvesting organoid samples for scRNA-seq or immunostaining, d19–d20 aggregates were pre-screened based on the epithelial PAX2nG fluorescence signals, and d60–d70 aggregates with the POU4F3nT reporter knockin were pre-screened based on the hair cell-specific POU4F3nT fluorescence signals. Except for genotypes unable to produce hair cells (e.g. CHD7S834F/S834F and CHD7KO/KO), low quality organoids with few PAX2nG-positive epithelial vesicle structures or few POU4F3nT-positive hair cells were generally not used from subsequent immunostaining or scRNA-seq experiments. Covariates associated these low quality organoid samples are not considered relevant to inner ear biology, as inner ear tissue were not present or rarely found in these unsuccessfully differentiated samples.
Blinding	The investigators were not blinded to the experimental conditions. The same investigators performed experiments and data analysis, which

Blinding	made blinding difficult. The use of unbiased clustering during scRNA-seq analysis helped to mitigate any potential bias from a lack of blinding.

Reporting for specific materials, systems and methods

We require information from authors about some types of materials, experimental systems and methods used in many studies. Here, indicate whether each material, system or method listed is relevant to your study. If you are not sure if a list item applies to your research, read the appropriate section before selecting a response.

Materials & experimental systems

n/a	Involved in the study
☐	☒ Antibodies
☐	☒ Eukaryotic cell lines
☒	☐ Palaeontology and archaeology
☒	☐ Animals and other organisms
☒	☐ Clinical data
☒	☐ Dual use research of concern

Methods

n/a	Involved in the study
☒	☐ ChIP-seq
☐	☒ Flow cytometry
☒	☐ MRI-based neuroimaging

Antibodies

Antibodies used

Antibody name (Species, clone, Supplier, catalog number, dilution rate)

ANXA4 (goat, N/A, R&D Systems, AF4146, 1:100)
CDH1 (mouse, 36, BD Biosciences, 610181, 1:200)
CHD7 (rabbit, D3F5, Cell Signaling, 6505S, 1:400 WB)
CHD7* (sheep, N/A, R&D Systems, AF7350, 1:50 IHC, 1:400 WB)
CHD7 (rabbit, N/A, Abcam, ab31824, 1:400 WB)
COL9A2 (rabbit, N/A, Sigma, HPA056316, 1:100)
DLX5 (rabbit, N/A, Novus Biologicals, NBP1-19547, 1:100)
EPCAM (mouse, 9C4, BioLegend, 324202, 1:200)
EPCAM conjugated to PE (mouse, 9C4, BioLegend, 324206, 1:200)
FBXO2 (mouse, E-9, Santa Cruz, sc-398111, 1:25)
Flag (mouse, M2, Sigma-Aldrich, F1804, 1:100 IHC, 1:400 WB)
GAPDH conjugated to HRP (mouse, GA1R, Invitrogen, MA515738HRP, 1:2000 WB)
GFP (mouse, 3E6, Thermo Fisher, A-11120, 1:100)
HOXB9 (rabbit, E7P5O, Cell Signaling, 27967S, 1:100)
MYO7A (rabbit, N/A, Proteus, 256790, 1:100)
MYO7A (mouse, C-5, Santa Cruz, sc-74516, 1:20)
NEFL (rabbit, N/A, Millipore, Ab9568, 1:200)
OCT4 conjugated to AF488 (rat, EM92, eBioscience, 53584182, 1:100)
PAX8 (rabbit, N/A, Abcam, AB97477, 1:100)
PCP4 (rabbit, N/A, Santa Cruz, sc-74816, 1:400)
phalloidin conjugated to AF488 (N/A, N/A, Thermo Fisher, A12379, 1:100)
POU4F3 (mouse, QQ8, Santa Cruz, sc-81980, 1:25)
S100B (rabbit, EP1576Y, Abcam, Ab52642, 1:100)
SIX1 (rabbit, D4A8K, Cell Signaling, 12891, 1:100)
SOX10 (mouse, 20B7, eBioscience, 14-5923-82, 1:50)
SOX2 (mouse, O30-678, BD Pharmingen, 561469, 1:200)
SOX2 (rabbit, N/A, Millipore Sigma, AB5603, 1:100)
SPARCL1 (goat, N/A, R&D Systems, AF2728, 1:100)
SSEA4 conjugated to AF594 (mouse, MC-813-70, BioLegend, 330414, 1:200)
TFAP2A (mouse, 3B5, DSHB, 3B5, 1:5)
Abbreviations: mAb, monoclonal antibody. HRP, horseradish peroxidase. AF488, Alexa Fluor 488. AF594, Alexa Fluor 594. IHC, immunohistochemistry. PE, phycoerythrin. WB, western blot.
* Unless otherwise noted, all anti-CHD7 IHC and WB experiments in this study used this antibody.

Antibody information is also listed in Supplementary Table 1 of the manuscript.

Validation

Previously published or manufacturer-validated antibodies were used. The validation references were provided in Supplementary Table 1. For each antibody, the company website provides validation data. We typically chose antibodies with good Western blot data.

Antibody: Validation assay, Validation tissue type (Validation reference)

ANXA4: Immunofluorescence, Mouse inner ear (Burns, Kelly et al. 2015)
CDH1: Immunofluorescence, Mouse inner ear organoids (Koehler, Mikosz et al. 2013)
CHD7 (Cell Signaling #6505S): Immunofluorescence, Western blot, and ChIP-seq, Mouse non-otic tissue and non-otic cell line (He, Marie et al. 2016)
CHD7 (R&D Systems #AF7350): Western Blot, Human non-otic cell line (Yamamoto, Takenaka et al. 2018)

CHD7 (Abcam # ab31824): Western Blot and ChIP-seq, Mouse and human non-otic cell lines (Engelen, Akinci et al. 2011)
COL9A2: Immunofluorescence, Mouse inner ear (Hartman, Durruthy-Durruthy et al. 2015)
DLX5: Immunohistochemistry, Human non-otic tissue (Bellessort, Le Cardinal et al. 2016)
EPCAM: Mass cytometry , Human non-otic tissue (Wagner, Rapsomaniki et al. 2019)
EPCAM (conjugated to PE): Mass cytometry, Human non-otic tissue (Wagner, Rapsomaniki et al. 2019)
FBXO2: Immunofluorescence, Human inner ear organoids (Koehler, Nie et al. 2017)
Flag: Immunofluorescence and Western blot, Mouse non-otic cell line (Chu, Zhang et al. 2020)
GAPDH (conjugated to HRP): Western Blot, Human non-otic cell line (Wei, Wu et al. 2020)
GFP: Immunofluorescence, Mouse non-otic tissue and non-otic cell line (Shao, Feng et al. 2009)
HOXB9: Immunofluorescence and Western blot, Human non-otic cell lines (Szatanek 7/9/2020)
MYO7A: Immunofluorescence, Human inner ear organoids (Koehler, Nie et al. 2017)
MYO7A: Immunofluorescence, Mouse inner ear (Grati and Kachar 2011)
NEFL: Immunofluorescence, Human inner ear organoids (Koehler, Nie et al. 2017)
OCT4 (conjugated to AF488): Immunofluorescence, Human inner ear organoids (Koehler, Nie et al. 2017)
PAX8: Immunofluorescence, Human inner ear organoids (Koehler, Nie et al. 2017)
PCP4: Immunofluorescence, Mouse inner ear (Burns, Kelly et al. 2015)
phalloidin (conjugated to AF488): Immunofluorescence, Human inner ear organoids (Koehler, Nie et al. 2017)
POU4F3: Immunofluorescence, Human inner ear organoids (Koehler, Nie et al. 2017)
S100B: Immunofluorescence, Human non-otic tissue (Duan, Li et al. 2020)
SIX1: Immunofluorescence, Human non-otic tissue (Xu, Li et al. 2022)
SOX10: Immunofluorescence, Mouse non-otic tissue (Ferletta, Uhrbom et al. 2007)
SOX2: Immunofluorescence, Human inner ear organoids (Koehler, Nie et al. 2017)
SOX2: Immunofluorescence, Mouse inner ear (Brown, Nelson et al. 2020)
SPARCL1: Immunofluorescence, Mouse inner ear (Burns, Kelly et al. 2015)
SSEA4 (conjugated to AF594): Immunofluorescence, Human inner ear organoids (Koehler, Nie et al. 2017)
TFAP2A: Immunofluorescence, Human inner ear organoids (Koehler, Nie et al. 2017)

Full reference citation information included in the supplementary information file.

Eukaryotic cell lines

Policy information about cell lines and Sex and Gender in Research

Cell line source(s)	The human embryonic stem cell line WA25, purchased from WiCell, was used in this study.
Authentication	The authenticity of the WA25 line has been validated with short tandem repeat (STR) profiling, karyotyping, and sterility and mycoplasma testing by WiCell and the certificate of these analyses has been provided.
Mycoplasma contamination	The WA25 line has been tested negative for Mycoplasma contamination.
Commonly misidentified lines (See ICLAC register)	No ICLAC line was used.

Flow Cytometry

Plots

Confirm that:

☒ The axis labels state the marker and fluorochrome used (e.g. CD4-FITC).

☒ The axis scales are clearly visible. Include numbers along axes only for bottom left plot of group (a 'group' is an analysis of identical markers).

☒ All plots are contour plots with outliers or pseudocolor plots.

☒ A numerical value for number of cells or percentage (with statistics) is provided.

Methodology

Sample preparation

For d20 WT vs. CHD7KO/KO scRNA-seq, 30 WT aggregates (PAX2nG) and 30 CHD7KO/KO aggregates (CHD7KO/KO PAX2nG) (as well as 10 WA25 control aggregates dissociated in separate tubes and wells) were washed three times with DPBS, three times with 1.1 mM EDTA, followed by resuspension in an accutase solution. 10–15 aggregates were transferred to each well of a Nunclon Sphera low-binding 6-well plate (Thermo Fisher) along with 3 mL of accutase. The plates were incubated at 37℃ 5% CO2 for 90 min with gentle trituration every 5–10 min with a wide-bore P1000 pipet tip. Dissociated cells were filtered through a 100 μm cell strainer and then a 40 μm cell strainer (Corning), and then centrifuged in 2 mL round-bottom tubes at 100 × g for 3 min. Cell pellets were resuspended in a DMEM:F12 solution (with HEPES, no phenol red; Thermo Fisher) supplemented with 10% FBS (Thermo Fisher) and 1:500 propidium iodide (Thermo Fisher) cell viability dye. GFP+ propidium iodide- cells were sorted into a DMEM:F12 (with HEPES, no phenol red) solution supplemented with 10% FBS on a SORP Aria FACS machine (BD Biosciences) for 1 h at Indiana University Flow Cytometry Resource Facility, using dissociated cells from d20 WA25 aggregates as negative control for gating.

For d20 WT PAX2nG+ and PAX2nG– scRNA-seq, the cell dissociation and FACS sorting were performed similarly, with the exception that 1:500 7-AAD (BioLegend) was used as a viability dye, and that both GFP+ and GFP– populations were separately collected for downstream scRNA-seq experiments.

To enrich hair cells and supporting cells from d70 WT (POU4F3nT PAX2nG) and CHD7KO/+ (CHD7KO/+ POU4F3nT PAX2nG) organoids, tissues containing vesicle structures harboring the POU4F3nT-positive hair cells were micro-dissected from the rest of the d70 aggregates with fine tweezers (Dumont) under a fluorescence stereomicroscope. The dissected d70 organoid tissues were dissociated and FACS sorted in a similar way as d20 organoids, with the exception that no viability dye was used. tdTomato+ and tdTomato– cell populations were collected in separate tubes for separate downstream scRNA-seq reactions.

For d70 WT (POU4F3nT PAX2nG) vs. CHD7KO/KO (CHD7KO/KO PAX2nG) scRNA-seq, the POU4F3nT FACS sorting strategy can no longer be used to enrich the otic epithelial cell types, as the CHD7KO/KO organoids do not generate any POU4F3nT+ hair cells. Therefore, while POU4F3nT fluorescence signals was still used as a guide for micro-dissection for the WT organoids, phase contrast live imaging was used for micro-dissection of the CHD7KO/KO organoids to enrich tissues containing vesicle morphological structures. After tissue dissociation, cells were stained with 1:100 PE-conjugated EPCAM antibody (BioLegend) by nutating at 4℃ for 40 min in dark. No viability dye was co-stained. After antibody staining, cells were washed twice prior to FACS sorting. Both PE-EPCAM+ and PE-EPCAM– populations were collected in separate tubes for separate downstream scRNA-seq reactions. For WT samples, as the POU4F3nT+ hair cells also express EPCAM on their cell surface, the hair cells exhibit both tdTomato and PE fluorescence signals, both of which are red. As such, the WT hair cells were FACS-isolated as a sub-population of PE-EPCAM+ cell. The POU4F3nT reporter is highly specific to the EPCAM-expressing hair cells, and the PE-EPCAM– population does not contain any POU4F3nT signals.

Instrument

SORP Aria (BD Biosciences)

Software

BD FACSDiva and FlowJo

Cell population abundance

Cell population abundance was determined in the BD FACSDiva software as follows: 30.5% d20 WT GFP+ cells, 21.7% d20 CHD7KO/KO GFP+ cells, 32.7% d20 WT GFP+ cells, 54% d20 WT GFP- cells, 4.5% d70 WT tdTomato+ cells, 95% d70 WT tdTomato- cells, 4.6% d70 CHD7KO/+ tdTomato+ cells, and 95.2% d70 CHD7KO/+ tdTomato- cells, 5.7% d70 WT EPCAM/tdTomato+ cells, 94.1% WT EPCAM/tdTomato- cells, 4.7% d70 CHD7KO/KO EPCAM+ cells, and 95.2% CHD7KO/KO EPAM- cells.

Gating strategy

FSC-A vs. FSC-H and SSC-A vs. SSC-H gates were used to exclude debris and select for single cells. GFP or tdTomato reporter cells were sorted based on fluorescent signal. Fluorescent gating was established by using dissociated wild type WA25 aggregates as a negative control. Positive cells were defined by signal that exceeded the negative control. For experiments designed to collect tdTomato positive hair cells, gating was established to collect the highest intensity 10-30,000 events to enrich the target population and reduce downstream noise from weakly positive off-target cells.

☒ Tick this box to confirm that a figure exemplifying the gating strategy is provided in the Supplementary Information.
